# Application of *Wickerhamomyces anomalus* in Simulated Solid-State Fermentation for *Baijiu* Production: Changes of Microbial Community Structure and Flavor Metabolism

**DOI:** 10.3389/fmicb.2020.598758

**Published:** 2020-11-27

**Authors:** Wenhua Wang, Guangsen Fan, Xiuting Li, Zhilei Fu, Xin Liang, Baoguo Sun

**Affiliations:** ^1^Laboratory of Food Microbiology and Enzyme Engineering, Beijing Advanced Innovation Center for Food Nutrition and Human Health, Beijing Technology and Business University, Beijing, China; ^2^Laboratory of Food Microbiology and Enzyme Engineering, School of Food and Health, Beijing Technology and Business University, Beijing, China; ^3^School of Light Industry, Beijing Technology and Business University, Beijing, China

**Keywords:** *Baijiu*, bioaugmentation, *Daqu*, flavor, microbial community, *Wickerhamomyces anomalus*

## Abstract

*Wickerhamomyces anomalus* is conducive to the synthesis of ester compounds in brewing the Chinese liquor *Baijiu*; esters are crucial for the quality of *Baijiu*. In this study, simulated solid-state fermentation for *Baijiu* production was used to explore whether artificial addition of *W. anomalus* could improve the flavor substance in *Baijiu*, and the underlying mechanisms. Two experimental groups were studied, in which *W. anomalus* Y3604 (Group A) and YF1503 (Group B) were added, respectively; in the control group (Group C), no *W. anomalus* was added. Adding strain Y3604 increased the content of esters in fermentation samples, especially ethyl acetate and ethyl caproate, and reduced the content of higher alcohols. Adding strain YF1503 had little effect on the ester content but decreased the content of higher alcohols. The diversity and abundance of prokaryotic genera in Group A and B samples were similar, but there were some differences compared with Group C. The correlations of genera in Group A or B samples were simple compared with group C. Although the predominant eukaryotic genera in the three groups were consistent, the abundance of each gene varied among groups. Based on our findings, bioaugmentation of *Baijiu* fermentation with *W. anomalus* will change the ethyl acetate content and cause changes in the levels of other flavor substances. We suggest that the changes in flavor substances caused by the addition of *W. anomalus* are mainly due to changes in the microbial community structure that result from the addition of *W. anomalus*.

## Introduction

*Baijiu* is a traditional alcoholic beverage with thousands of years of history in China, and it occupies a unique position in Chinese traditional culture as a pearl of ancient Chinese wisdom (Han, 2016; Jin et al., 2017; Sun et al., 2018a; Song et al., 2020). The quality of *Baijiu* is highly influenced by its special flavor and mouthfeel in the mouth, a determinant factor in acceptance of *Baijiu* (Fan et al., 2018b). Volatile compounds play key roles in the flavor of *Baijiu* (Li et al., 2019), which contains > 1870 flavoring compounds (Liu and Sun, 2018). Because of their low odor thresholds and synergistic effects, esters, with fruit aroma, fragrance, and sweet smell, are the major contributors to the aroma of *Baijiu*; minor changes in their concentrations can have significant effects on the flavor of the beverage (Fan and Qian, 2006; Chen and Xu, 2010; Li et al., 2017; Fan et al., 2018b). The quantity and proportion of esters are important indicators to distinguish different quality grades of *Baijiu* (Fan et al., 2018b). Ethyl acetate, ethyl lactate, ethyl caproate, and ethyl butyrate have been identified as the main four ethyl esters (MFEEs) in *Baijiu*, and ethyl acetate is the principal indicator of quality for various types of *Baijiu* in almost all standards (Fan et al., 2018b).

During *Baijiu* production, ethyl acetate comes from three sources: introduction into the system along with raw material; reaction of acetic acid with ethanol; and microbial metabolism. Microbial metabolism is the main source of ethyl acetate in *Baijiu* manufacture (Fan et al., 2019b). Many strains, including bacteria, molds and yeasts, produce ethyl acetate in the *Baijiu* production process (Guo and Jia, 2014; Fan et al., 2019b,c). Previous studies have shown that *Wickerhamomyces anomalus* is the main species producing ethyl acetate, so *W. anomalus* strains make a special contribution to *Baijiu* quality (Fu et al., 2018; Fan et al., 2018b, 2019b,c). Our research team has access to two strains of *W. anomalus*, named Y3604 and YF1503, that are reported to be among the best strains for ethyl acetate production (Fu et al., 2018; Fan et al., 2018b). They enhance production of ethyl acetate when they are co-cultured with *Saccharomyces cerevisiae* in a liquid medium (Fan et al., 2019b, c). However, the production process of *Baijiu* is more complicated than culture in liquid medium—*Baijiu* production is a spontaneous process involving complex communities of microorganisms (Liu and Sun, 2018). Studying only the relationship between *W. anomalus* and *S. cerevisiae* in liquid fermentation is far from enough to show that *W. anomalus* has good effects in the process of *Baijiu* production. This is because *Baijiu* production environment and the relationship with many other microbial communities will affect *Baijiu* production process. This study adopted the method of simulated solid-state fermentation (SSF) to explore the effects of *W. anomalus* Y3604 and YF1503 in *Baijiu* production and aimed to improve the content of ethyl acetate in raw *Baijiu* (no blending) to improve its quality. We determined the effects of the *W. anomalus* strains in *Baijiu* production by analyzing changes of microbial community structure and flavor metabolites.

## Materials and Methods

### Strains and Materials

*Wickerhamomyces anomalus* Y3604 and YF1503 were isolated from *Daqu* and deposited in the China General Microbiological Culture Collection Center under accession numbers 13103 and 14169, respectively (Fu et al., 2018; Fan et al., 2018b). *Daqu* samples, stored for 3 months, were collected from Beijing Shunxin Agriculture Co., Ltd., Beijing, China, in April 2018.

### Solid-State Fermentation

Solid-state fermentation was performed to study the application of two *W. anomalus* yeasts. SSF was established on the basis of *Baijiu* production process according to a previous report with slight modifications (Fan et al., 2020). The process is shown in Supplementary Figure S1. *W. anomalus* strains Y3604 and YF1503 were inoculated into yeast extract peptone dextrose (YPD) liquid medium at 30°C and cultured for 24 h with shaking at 180 rpm. Cells were harvested by centrifugation at 2,348 × *g* for 10 min and washed three times with 0.9% normal saline to prepare cell suspensions. Three groups were prepared per the method in the previous report: Group A: *Daqu* with an initial density of 1 × 10^6^ colony-forming units (CFU) of *W. anomalus* strain Y3604/mL; Group B: *Daqu* with an initial density of 1 × 10^6^ CFU *W. anomalus* strain YF1503/mL; and Group C (the control group): *Daqu* with 0.9% normal saline solution instead of a strain suspension (Fan et al., 2020). A mixture of cracked sorghum (200 g) and rice husk (50 g) was immersed in hot water (80°C) for 24 h, then steamed for 1.5 h and cooled to room temperature. *Daqu* (40 g) and strain suspension (10 mL) were added, and the mixture was transferred to a 1.5-L ceramic jar (the moisture content was about 50%). Each group was tested in two separate batches. Samples were collected after 0, 6, 12, 18, and 24 days, and kept at −80°C until analysis.

### Volatile Compound Analysis

Volatile compounds in SSF samples were detected by headspace solid-phase microextraction gas chromatography–mass spectrometry (HS-SPME-GC-MS) with a TraceGC TSQ 8000 Evo instrument (Thermo Fisher Scientific, Waltham, MA, United States), according to a previously reported method (Fan et al., 2018a). Each volatile compound was identified and quantified by comparing the acquired spectrum with mass spectral data in the NIST 05a library (Thermo Fisher Scientific) and using the response for the internal standard 2-octanol (Sigma-Aldrich, St. Louis, MO, United States), respectively. Each volatile compound concentration is reported as the mean concentration found for two analyses in milligrams per kilogram of SSF sample.

### Microbial Community Structure Analysis

Genomic DNA was extracted from treated samples per the report of Zhang et al. using a Power Soil DNA Isolation Kit (Mo-Bio, Carlsbad, CA, United States) (Zhang et al., 2014). After detection by electrophoresis with a 0.6% agarose gel, the required DNA samples were stored at -80°C. The prokaryotic microbiota were analyzed by amplifying the V3–V4 hypervariable region of 16S rRNA genes with primers 340F (5′-barcode-CCTACGGGNBGCASCAG-3′) and 805R (5′-GACTACNVGGGTATCTAATCC-3′). Primers 512F (5′-TATTCCAGCTCCAATAGCG-3′) and 978R (5′-barcode-GACTACGATGGTATCTAATC-3′) were used to amplify the 18S rRNA region for analysis of the eukaryotic community. The amplification was performed following a published method (Fan et al., 2020). The amplified products were analyzed, purified, and quantified using agarose gel electrophoresis, the AxyPrep DNA Gel Extraction Kit (Axygen Biosciences, Union City, CA, United States), and QuantiFluor-ST (Promega Corporation, Madison, WI, United States), respectively. After passing the quality inspection, they were pooled in equal amounts for pair-end (2 × 250 bp) sequencing on an Illumina HiSeq2500 sequencing platform (Illumina, San Diego, CA, United States) in accordance with the standard procedure. The raw sequences were processed with QIIME (version 1.8). Sequences that could not be assembled were trimmed, and chimeric sequences were removed using UCHIME. After alignment and merging using FLASH, sequences were clustered into operational taxonomic units (OTUs) using UPARSE version 7.1^1^ at a 97% identity threshold. Using the Ribosomal Database Project Classifier^2^ and the Silva (SSU115) 16S rRNA and 18S rRNA database, the taxonomic classification of each 16S rRNA/18S rRNA gene sequence was determined at a confidence level of 70%. Alpha diversity indices, including Good’s coverage, Chao1, ACE, and Shannon and Simpson indices, and beta diversity were calculated using the QIIME suite of programs and assessed using the UniFrac method, respectively. The similarities of different SSF samples were evaluated by performing UniFrac analyses. Principal coordinate analysis (PCoA) was achieved by performing weighted and unweighted calculations. Correlations between community structures and environmental variables were evaluated by performing redundancy analysis (RDA) using Vegan software, and a heatmap was constructed using the heatmap package in R software^3^. The raw reads were submitted to the NCBI Sequence Read Archive (SRA) database (accession number PRJNA658434).

### Statistical Analysis

Statistical analyses were performed using SPSS software v. 17.0 (SPSS Inc., Chicago, IL, United States).

## Results and Discussion

### Volatile Compounds

**FIGURE 1:**
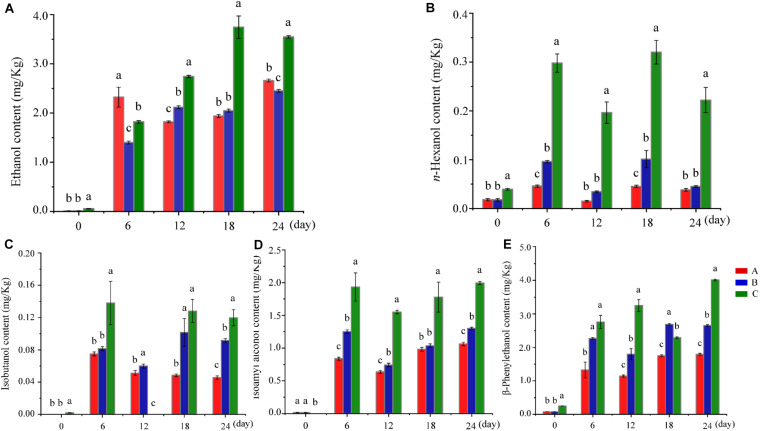
Changes in ethanol and higher alcohols in different groups during fermentation. **(A–E)** Shows the ethanol content, *n*-hexanol content, isobutanol content, isoamyl alcohol content, and β-phenylethanol content at various fermentation stages in different groups, respectively. Different letters indicate significant differences (*P* < 0.05). Red bar, blue bar and green bar represents Group A, B and C, respectively.

Forty-four volatile compounds (six alcohols, thirty esters, three aldehydes, three phenols, and two acids) in the fermentation samples were identified by HS-SPME-GC-MS (Supplementary Table S1). Supplementary Table S1 also shows the changes of flavor substances. The types and contents of flavor substances in the same group changed with the extension of fermentation time, which is mainly caused by metabolism of microorganisms and chemical reactions among flavor substances. However, more attention should be paid to differences in the types and contents of flavor substances in different groups at the same fermentation time. The apparent reason is the addition of *W. anomalus*, but the mechanism may be the metabolic action of the changed microbiotic community and chemical reactions among the different flavor substances generated after the change of microbiota caused by adding *W. anomalus*. In view of the main purpose of this study, we will not analyze the changes of flavor substances in the same group at different fermentation times, but will compare and analyze the changes of flavor substances in different experimental groups at the same fermentation time, as follows:

Ethanol content determines the production of *Baijiu*. Compared with Group C (the control group), a low concentration of ethanol was presented in Groups A and B, even though Group A contained a relatively high amount of ethanol in the early stage of fermentation (day 6). The ethanol content of Group C continuously increased from the middle stage of fermentation (day 12), peaked on day 18, and remained relatively stable until the end of fermentation (Figure 1A). In other words, adding *W. anomalus* led to a decrease of *Baijiu* production, which was similar to the effect of adding other non-*Saccharomyces* strains (Canonico et al., 2016). Some of the mechanisms responsible for reduced ethanol yields include altered biomass synthesis, reduction of the biomass or vitality of *S. cerevisiae*, by-product (such as ester) formation, and/or alternative regulation of metabolisms caused by the change of microbiota structure and interaction in the fermentation process (Ciani et al., 2016). Higher alcohols, including *n*-propanol, *n*-hexanol, isobutanol, isoamyl alcohol, and β-phenylethanol, are a large group of monovalent alcohols with more than three carbon atoms and are flavor compounds in many alcoholic beverages (Styger et al., 2011; Pires et al., 2014). They have a significant effect on the sensorial quality and character of alcoholic beverages because of their strong, pungent smell and taste (Swiegers and Pretorius, 2005). With appropriate higher alcohols content, the beverages will be are mellow, soft, plump, and have a pleasant bouquet; however, if the content of higher alcohols is above a certain threshold, the beverage will have a fusel oil taste, be strongly intoxicating, and potentially be harmful to the human body (Zhang et al., 2015). Therefore, controlling the content of higher alcohols in *Baijiu* is vital for improving the position and expanding the market of *Baijiu* (Zhang et al., 2015). As shown in Figures 1B–E and Supplementary Table S1, the amount of higher alcohols in Groups A and B (to which *W. anomalus* had been added) was lower than that in the control group (Group C) as ethanol content. Generally, yeast, including *S. cerevisiae* and non-*Saccharomyces* strains, can produce higher alcohols in two ways: a biosynthetic route from a carbon source (Dickinson and Norte, 1993), or by degradation of branched-chain amino acids via the Ehrlich pathway (Derrick and Large, 1993; Hazelwood et al., 2008). Previous studies have shown that *W. anomalus* can produce a high content of higher alcohols (Oro et al., 2018). However, the results of the present study showed that the addition of either of the two tested strains of *W. anomalus* did not cause the higher alcohol content to increase, but to decrease. It may be that the structure of the microbiota was changed after addition of these yeasts, which resulted in changes in metabolic pathways, or promoted the conversion of higher alcohols into other flavor substances such as esters.

**FIGURE 2:**
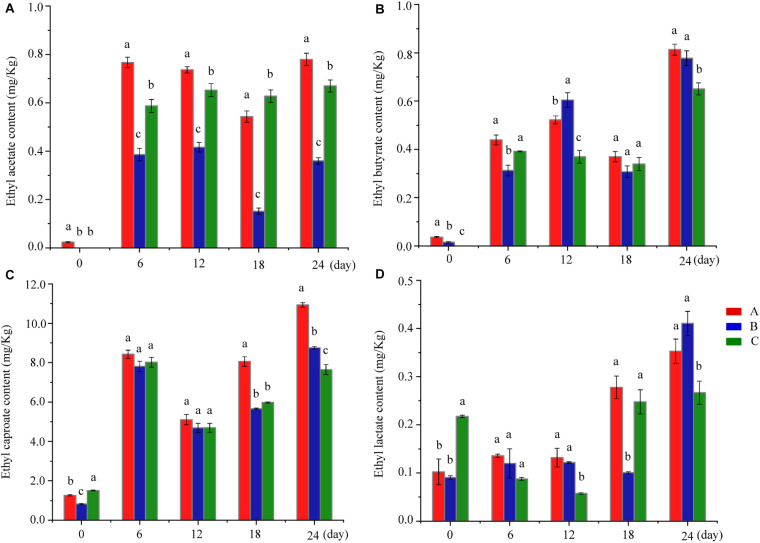
Changes in four ethyl esters in different groups during fermentation. **(A–D)** Shows the ethyl acetate content, ethyl butyrate content, ethyl caproate content and ethyl lactate content at various fermentation stages in different groups, respectively. Different letters indicate significant differences (*P* < 0.05). Red bar, blue bar, and green bar represents Group A, B, and C, respectively.

The quality of *Baijiu* depends on the quantity and proportion of esters. Esters produce the main characteristics of *Baijiu*, including the flavor, aroma, quality, grade, and the style of the product, which differ from those of other distillates (Fan and Qian, 2006; Kurita, 2008; Sumby et al., 2010; Li et al., 2017). The MFEEs content ranges from 90 to 95% of the total esters, and these have been identified as the main aroma components in *Baijiu* (Fan et al., 2018b). As shown in Figure 2 and Supplementary Table S1, compared with the control group, the MFEEs content increased in Group A. In Group A, the increase of ethyl acetate is probably related to the addition of *W. anomalus* Y3604, which converts ethanol into ethyl acetate (Fan et al., 2018b). Of course, it cannot be excluded that these changes arise from changes in other microbiota with high esterification activity caused by the addition of *W. anomalus* Y3604 to the fermentation. The increase in the other three of the MFEEs, which surprised us, is more likely due to changes in microbiota during fermentation caused by addition of *W. anomalus* Y3604, which may be conducive to the increase of microbiota with high esterifying-enzyme activity. Compared with control samples, ethyl butyrate, ethyl caproate, and ethyl lactate increased, while ethyl acetate showed a downward trend in Group B, which is not conducive to the quality of *Baijiu* (Han, 2016). The changes of MFEEs in Groups A and B showed some differences, which indicates that the two strains of *W. anomalus* have different characteristics and functions, as reported in previous studies (Fu et al., 2018; Fan et al., 2018b). Based on the high ethyl acetate production activity of *W. anomalus* YF1503 and the change of content of ethyl acetate in Group B samples, we speculate that the main reason for the change of ester compounds during *Baijiu* brewing in Group B may be change in the microbiota following addition of the *W. anomalus* strain. In addition, levels of ethyl caprate and ethyl octanoate, which give a fruit aroma, were lower in Group A than those in Group C, while no significant change was observed in Group B compared with Group C (Supplementary Table S1). The level of ethyl heptanoate, which has a fruit aroma, was higher in Groups A and B than in Group C, while phenethyl acetate, which contributes floral odors to *Baijiu*, was less abundant in Groups A and B than in Group C (Sha et al., 2017; Zhao et al., 2018; Sun et al., 2018b; Li et al., 2019). The main reason for the different content of esters in the different groups is the change in the microbiotic communities of the fermentation cultures resulting from the addition of the *W. anomalus* strain.

Phenolic compounds are important aromatic compounds in *Baijiu* (Han, 2016). Phenolic compounds, which are natural antioxidants, have attracted considerable attention for *Baijiu* production because of their possible beneficial effects on human health (Zhao et al., 2017). In Group A, the content of guaiacol (aromatic, roast) changed little, but the content of 4-ethylguaiacol (clove) decreased, and the content of 2-methoxy-4-vinylphenol ethylguaiacol (phenolic, clove-like odors) increased compared with control samples (Supplementary Table S1) (Zhang et al., 2013; Xiao et al., 2017; Lin et al., 2018; Zhao et al., 2018; Dong et al., 2019; Zhu et al., 2020). In Group B, the content of 2-methoxy-4-vinylphenol ethylguaiacol did not change, but the content of the other two phenols decreased relative to the controls. The effects of the two strains of *W. anomalus* on *Baijiu* brewing differ; this may be caused by different changes they induce in the microbiota in the brew.

### Prokaryotic Community Structure and Diversity

An average of 56,774 effective sequences were obtained from 30 samples after quality control. All the rarefaction curves based on observed species were saturated, which indicated that adequate sampling and sequencing had been performed (Supplementary Figure S2a). In Group A, the richness (indicated by the Sobs and Chao1 indexes; Table 1) increased in the early stage of the fermentation (days 0–6), decreased then increased in the middle stage (days 6–18), then remained stable (days 18–24). The diversity (indicated by the Shannon and Simpson indexes; Table 1) decreased in the early stage (days 0–6), increased in the middle stage (days 6–18), then decreased in the final stage (days 18–24). In Group B, the richness decreased in the early stage (days 0–6), increased in the middle stage (days 6–18) and remained unchanged thereafter. However, the diversity of samples in Group B fluctuated. In Group C, the richness increased in the early stage (days 0–6) then decreased (days 6–24). The change of diversity in Group C was similar to that in Group A (Table 1). In general, the richness and diversity in Groups A and B were higher than those in Group C, which was consistent with previous reports (He et al., 2020). That is, after the addition of *W. anomalus* Y3604 or YF1503, the prokaryotic community structure changed during SSF compared with that in the control group without adding *W. anomalus*. In addition, although the Wilcoxon rank-sum test for the Shannon and Simpson indexes indicated that the diversity differed significantly between Groups A or B and C (Supplementary Figures S3a,b), the changes of richness and diversity in Groups A and C were similar (compared with those in Group B), which indicates that the changes in the prokaryotic community structure caused by adding strain YF1503 were more intense than those caused by adding strain Y3604 (Table 1).

**TABLE 1:** Summary of sequencing results and the alpha diversity statistical analysis of samples from the three groups.

Type	Sample ID	Sobs	Shannon	Simpson	Chao1	Coverage
Prokaryotic	A0	151.5	2.64	0.18	161.00	0.9997
	A6	180.0	2.13	0.27	194.80	0.9997
	A12	151.0	2.42	0.22	156.00	0.9998
	A18	165.5	2.55	0.21	171.31	0.9999
	A24	168.0	1.67	0.42	176.31	0.9997
	B0	187.5	2.33	0.21	205.81	0.9996
	B6	128.0	2.97	0.12	131.50	0.9999
	B12	160.5	1.76	0.38	169.68	0.9998
	B18	161.0	2.68	0.20	166.21	0.9999
	B24	163.0	1.97	0.33	177.50	0.9997
	C0	142.0	1.78	0.32	151.48	0.9997
	C6	186.0	1.55	0.51	192.53	0.9997
	C12	181.0	1.27	0.53	211.00	0.9993
	C18	147.0	1.37	0.56	152.92	0.9998
	C24	142.5	1.21	0.59	165.50	0.9996
Eukaryotic	A0	24.5	1.71	0.24	24.63	1.0000
	A6	22.0	0.84	0.64	23.67	0.9999
	A12	20.0	1.21	0.42	20.75	1.0000
	A18	20.5	1.23	0.39	25.50	0.9999
	A24	20.5	1.28	0.35	23.00	0.9999
	B0	31.0	1.84	0.22	32.92	0.9999
	B6	23.5	1.67	0.25	23.67	1.0000
	B12	19.5	1.49	0.29	24.00	0.9999
	B18	19.5	1.61	0.24	22.50	0.9999
	B24	20.0	1.57	0.25	21.17	0.9999
	C0	32.0	1.61	0.28	32.38	0.9999
	C6	19.5	0.54	0.78	23.88	0.9999
	C12	16.5	0.31	0.89	20.63	0.9999
	C18	16.0	0.31	0.89	16.00	1.0000
	C24	18.5	0.23	0.92	23.38	0.9999

*The samples taken from different experimental groups at different days were labeled as a combination of corresponding groups and days.*

**FIGURE 3:**
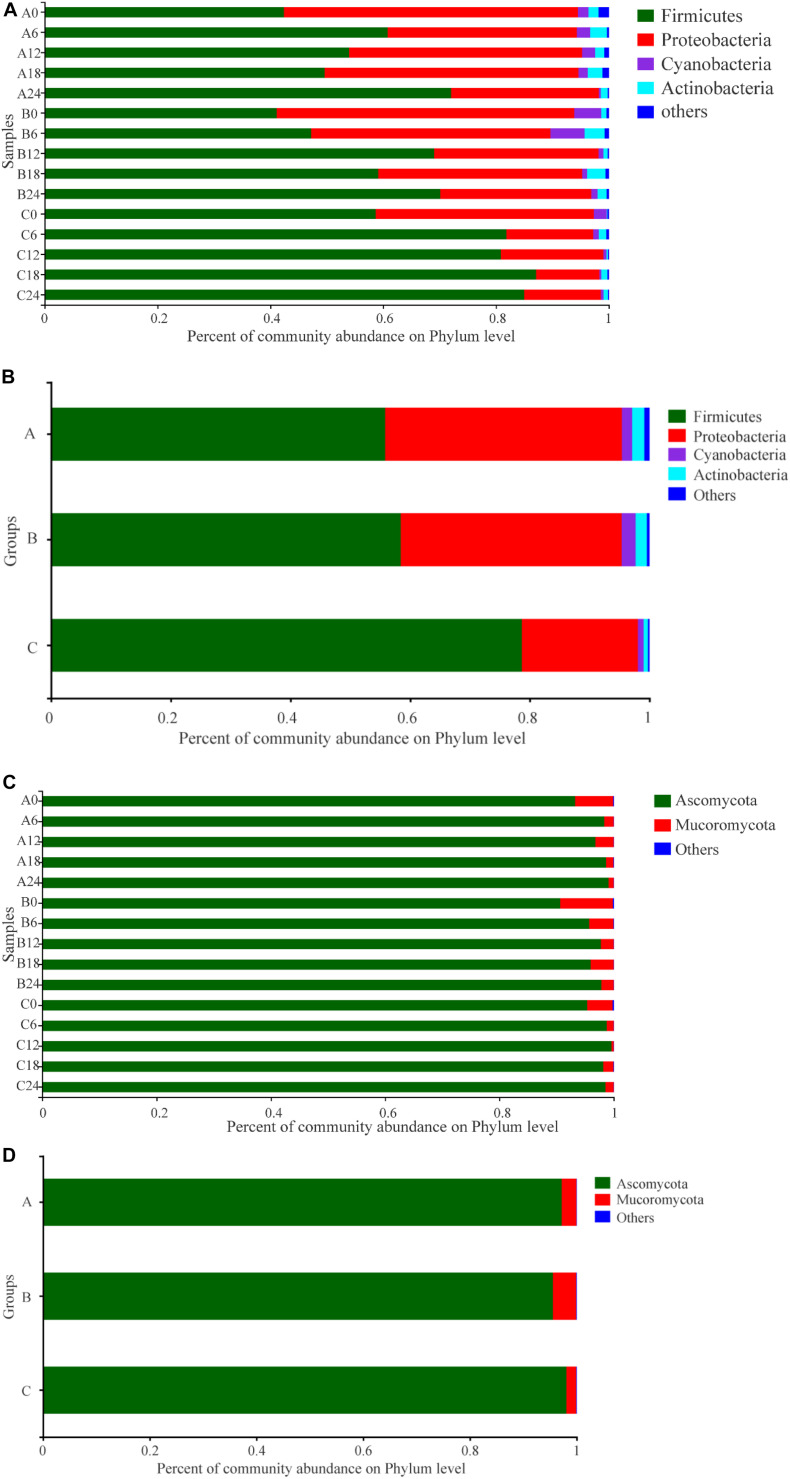
Distribution of the relative abundances of prokaryotic **(A,B)** and eukaryotic **(C,D)** microbiota at the phylum level. The relative abundance defines sequence percentages in samples as depicted by the colors in the bar chart. Only those phylum that had an average abundance greater than 1% are indicated, phylum with less than 1% abundance are combined and shown in “others” category. Analysis of prokaryotic microbiota composition in different groups during fermentation are shown in **(A,B)**. Results of eukaryotic microbiota in different groups at the phylum level are shown in **(C,D)**. The samples taken from different experimental groups at different days were labeled as a combination of corresponding groups and days. For example, A6 represents the sample on the 6th day of fermentation in experimental Group A.

The taxonomic distributions of prokaryotes in the samples at the phylum level are shown in Figures 3A,B. A total of 23 prokaryotic phyla were found in these samples: 17 in Group A, 18 in B, and 17 in C. Among these, the predominant phyla were Firmicutes, Proteobacteria, Cyanobacteria, and Actinobacteria, which was in accordance with previous reports (Fan et al., 2019a). In Group A, the abundance of Firmicutes increased in the early stage (days 0–6), decreased in the middle stage (days 6–18), then increased in the final stage (days 18–24). The change trend of Proteobacteria was the opposite to that of Firmicutes. The abundance of Cyanobacteria increased in the early stage, then decreased. In Group B, the change trends of Firmicutes and Proteobacteria were similar to those in Group A, but there was a slight difference in the timing. In Group C, the abundance of Firmicutes first increased then stabilized; Proteobacteria and Cyanobacteria showed a decrease then stabilized. In addition, the change of Actinobacteria was frequent, but the change pattern was similar in all groups. Meanwhile, throughout the SSF process, the abundance of Firmicutes in Groups A and B was lower than that in Group C, while the abundance of Proteobacteria, Cyanobacteria, and Actinobacteria in Groups A and B was higher than that in Group C. It can be seen that there was a difference in the prokaryotic communities at the phylum level compared with controls after addition of *W. anomalus*, and there were also some differences between Groups A and B, to which different strains of *W. anomalus* were added.

**FIGURE 4:**
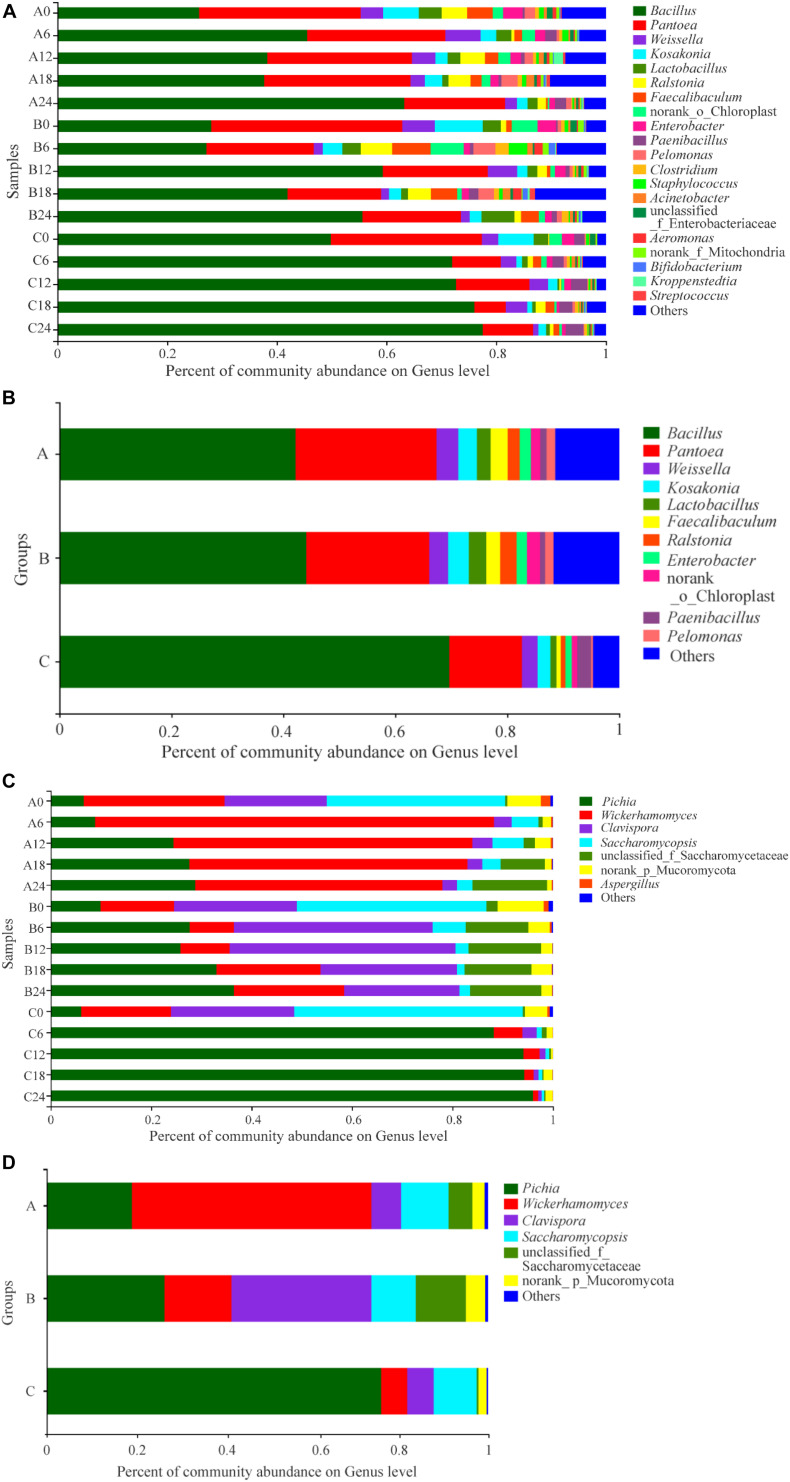
Distribution of the relative abundances of prokaryotic **(A,B)** and eukaryotic **(C,D)** microbiota at the genus level. The relative abundance defines sequence percentages in samples as depicted by the colors in the bar chart. Only those genus that had an average abundance greater than 1% are indicated, genus with less than 1% abundance are combined and shown in “others” category. Analysis of prokaryotic microbiota composition in different groups during fermentation are shown in **(A,B)**. Results of eukaryotic microbiota in different groups at the genus level are shown in **(C,D)**. The samples taken from different experimental groups at different days were labeled as a combination of corresponding groups and days.

**FIGURE 5:**
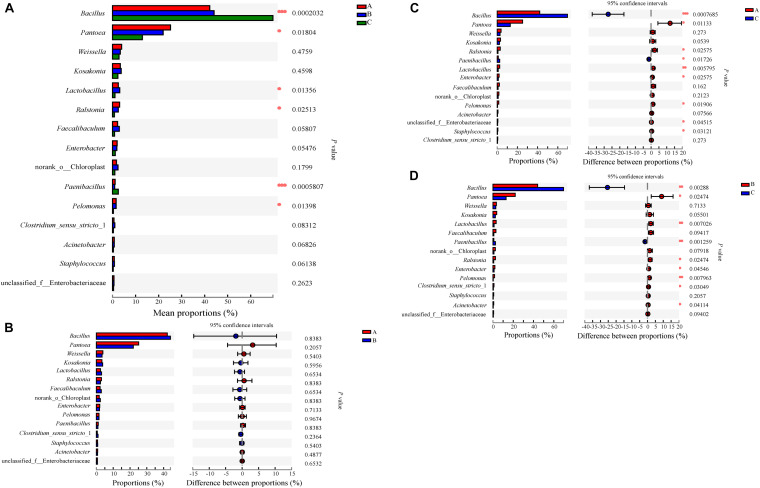
Relative abundances of prokaryotic microbiota that showed significant differences among three groups at genus level. Data of groups were showed as relative abundance of genus in each group. **(A)** A one-way ANOVA was used to evaluate the significance of differences among three groups; **(B–D)** showed that phylotypes significantly different between Groups A and B, Groups A and C, and Groups B and C, respectively, at genus level, and statistical analysis was performed by the Wilcoxon rank-sum test. Asterisk shows significant differences (“*”, 0.01 < *P* < 0.05; “**”, 0.001 < *P* < 0.01; “***”, *P* < 0.001).

A total of 417 abundant genera were found in SSF samples: 261 in Group A, 258 in Group B, and 268 in Group C (Supplementary Figure S4a). There was no significant difference in the number of genera among the groups. Venn analysis indicated that 172 genera were found in both Groups A and B, 169 genera were found in both Groups A and C, 172 genera were found in both Groups B and C, and 143 genera were found in all three groups (Supplementary Figure S4a). The dominant genera with relative abundances > 1% in Groups A and B were similar; they were *Bacillus* (which contributed 42.19 and 44.10% of the sequences in Groups A and B, respectively), *Pantoea* (25.15% in A, 21.96% in B), *Weissella* (3.90% in A, 3.38% in B), *Kosakonia* (3.32% in A, 3.72% in B), *Lactobacillus* (2.45% in A, 3.11% in B), *Ralstonia* (3.03% in A, 2.45% in B), *Faecalibaculum* (2.19% in A, 2.92% in B), *Enterobacter* (1.97% in A, 1.88% in B), norank_o_Chloroplast (1.68% in A, 2.34% in B), and *Pelomonas* (1.55% in A, 1.53% in B) (Figures 4A,B). In addition to the above genera, *Paenibacillus* was a dominant genus in Group A (1.12%), but only accounted for 0.90% of sequences in Group B. Compared with Groups A and B, the predominant genera in Group C were somewhat different in type and content; the dominant genera in Group C were *Bacillus* (69.63%), *Pantoea* (12.97%), *Weissella* (2.82%), *Kosakonia* (2.30%), *Lactobacillus* (1.05%), *Enterobacter* (1.13%), and *Paenibacillus* (2.47%). The abundance of *Bacillus* or *Paenibacillus* was lower in Groups A or B than in Group C at the same fermentation time throughout the process, while *Pantoea*, *Kosakonia*, *Lactobacillus*, *Enterobacter*, and *Pelomonas* were more abundant in Groups A or B than in Group C. One-way analysis of variance of the results showed that, at the phylum level, the abundance of Firmicutes, Proteobacteria, Actinobacteria, Bacteroidetes was different or significantly different in the three groups (Supplementary Figure S5). We further compared the relative levels of microbiota among them. Comparative analysis showed that these differences are between Groups A or B and Group C; there were no differences between Groups A and B (Supplementary Figure S5). At the genus level, the abundance of *Bacillus*, *Paenibacillus*, *Pantoea*, *Lactobacillus*, *Ralstonia*, and *Pelomonas* showed differences or significant differences in the three groups (Figure 5). Again, these differences were between Groups A or B and Group C. Therefore, there were some differences between the experimental groups and the control group in the composition and proportion of the dominant prokaryotic taxa. In fact, in addition to the different compositions and proportions of genera from the whole view, more importantly, the pattern of change of some genera during the SSF process differed among groups. For example, the abundance of *Bacillus*, which was the dominant genus consistent with previous reports (He et al., 2019a), increased in the early stage (days 0–6), decreased in the middle stage, and increased in the final stage of fermentation in Group A; in Group B, it was stable in the early stage, increased then decreased in the middle stage, and increased in the final stage; while in Group C, it increased in the early stage then remained stable (Figures 4A,B). The abundance of *Pantoea*, which was the second most dominant genus, was stable in Group A throughout the process compared with Group C, only decreasing at the end; in Group B, after decreasing in the early stage, it was more stable than in Group C. Lactic acid bacteria (LAB) genera are one of the dominant bacterial groups in *Baijiu* fermentation (Fan et al., 2019a). LAB play a significant role in improving *Baijiu* quality, because they can produce lactic acid for esterification by yeasts and also influence the microbial community structure (Yousif et al., 2010). Five LAB genera were identified in these samples: *Weissella*, *Lactobacillus*, *Faecalibaculum*, *Bifidobacterium*, and *Streptococcus*. The abundance of LAB in Groups A and B was higher than that in Group C, which may be a reason for the relatively high content of esters, especially ethyl lactate, in Groups A and B. However, the abundance of LAB decreased throughout the process in Group A, while in Groups B and C, it fluctuated with fermentation time. Notably, no *Bifidobacterium* or *Streptococcus* were detected in Group A samples, but they were detected in Groups B and C. Even where the same genera were detected in different samples, their patterns of change differed because of the addition of *W. anomalus*, which led to different flavor compositions of the *Baijiu*. He et al. (2019a) reported that bioaugmentation with *Bacillus* would alter the indigenous microbial community and subsequently improve the flavor characteristics of *Daqu*.

**FIGURE 6:**
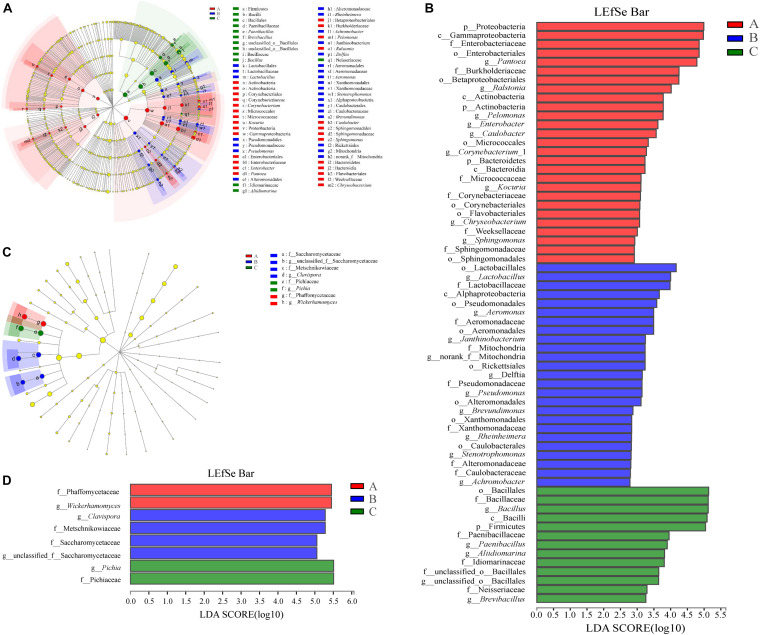
Prokaryotic **(A,B)** and eukaryotic **(C,D)** microbiota difference identification in three groups. **(A,C)** Taxonomic representation of statistically and biologically consistent differences among three groups with different treatments. Differences are represented by the color of the most abundant class (red, Group A; blue, Group B; green, Group C; yellow, non-significant). Circles indicate phylogenetic levels from phylum to genus. The diameter of each circle is proportional to the abundance of the group. **(B,D)** Linear discriminant analysis score, used to build the cladogram among three groups. Histogram of the LDA scores for indicator prokaryotic microbiota with LDA scores of 2 or greater in prokaryotic communities. LDA scores were calculated by LDA effect size, using the linear discriminant analysis to assess effect size of each differentially prokaryotic taxa (red, Group A; blue, Group B; green, Group C). The cladogram is displayed according to effect size.

The groups were represented in cladograms, and linear discriminant analysis (LDA) scores of ≥ 2 were confirmed by linear discriminant analysis effect size (LEfSe) (Figures 6A,B). In Group A, six groups of bacteria were significantly enriched compared with other two groups, namely Bacteroidetes (the phylum and genus *Chryseobacterium*), Sphingomonadales (the order and genus *Sphingomonas*), Proteobacteria (the phylum and genera *Pelomonas* and *Ralstonia*), Enterobacteriales (the order and genera *Enterobacter* and *Pantoea*), Actinobacteria (the phylum and genus *Kocuria*), and Corynebacteriales (the order and genus *Corynebacterium*_1). In Group B, seven groups of bacteria were significantly enriched compared with Groups A and C, namely Alphaproteobacteria (the class and its family Mitochondria to genus), Alphaproteobacteria (the class and genus *Brevundimonas*), Aeromonadales (the order and genus *Aeromonas*), Xanthomonadales (the order and genus *Stenotrophomonas*), Alteromonadales (the order and genus *Rheinheimera*), Pseudomonadales (the order and genus *Pseudomonas*), and Lactobacillales (the order and genus *Lactobacillus*). In Group C, only Firmicutes (the phylum and genera *Paenibacillus* and *Bacillus*) had levels comparable to those in Groups A and B. To evaluate the extent of the differences in the bacterial communities, weighted UniFracPCoA at the genus level was employed; this indicated an obvious separation of Groups A and B from Group C, while there was no obvious separation between Groups A and B (Supplementary Figure S6). Meanwhile, samples in Group C were well clustered, but samples in Groups A and B were dispersed and interweaved with each other with the advance of fermentation time. This further indicated that the bacterial community structure in the SSF changed after addition of *W. anomalus* (compared with the control), which led to differences in production of flavor substances. Previous reports showed that the inoculation of extrinsic microbes would inevitably bring about changes to the indigenous microbial community (Song et al., 2019).

**FIGURE 7:**
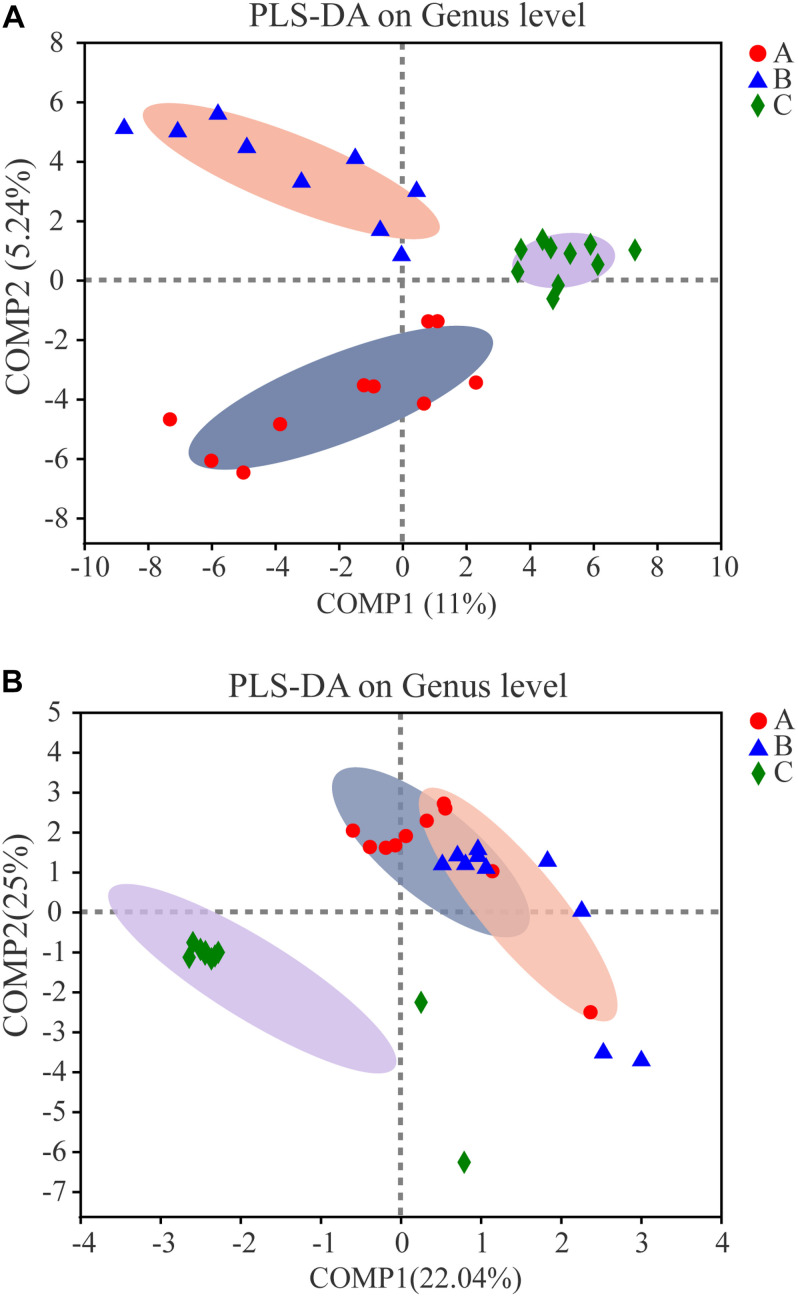
Partial least square discriminant score plot of microbiota at genus level among three groups. Red dots, blue triangular and green diamond represent Group A, B and C, respectively. **(A)** For prokaryotic microbiota; **(B)** for eukaryotic microbiota.

The bacterial communities in the samples from the three groups clustered separately by partial least squares discriminant analysis (PLS-DA), which suggests that the overall structures of the communities in the groups were significantly different (Figure 7A). Spots representing Group A or B samples presented more dispersed distribution patterns than those for the controls, which aligned with the complexity of bacterial diversity found in the Group A or B samples. The calculated *P*-values (*P* = 0.003 for Adonis, *P* = 0.002 for ANOSIM) by non-parametric multivariate analysis of variance (Adonis) and an analysis of similarities (ANOSIM) further demonstrated the differences among the bacterial communities in the groups. Enterotype analysis indicated that almost all samples belonged to the *Bacillus* enterotype, but some samples from Groups A and B belonged to the *Pantoea* enterotype, which indicates that samples in Groups A and B changed compared with controls (Group C) because of the addition of *W. anomalus*. Addition of *W. anomalus* is beneficial for the activity of *Pantoea* in the SSF (Supplementary Figure S7a).

**FIGURE 8:**
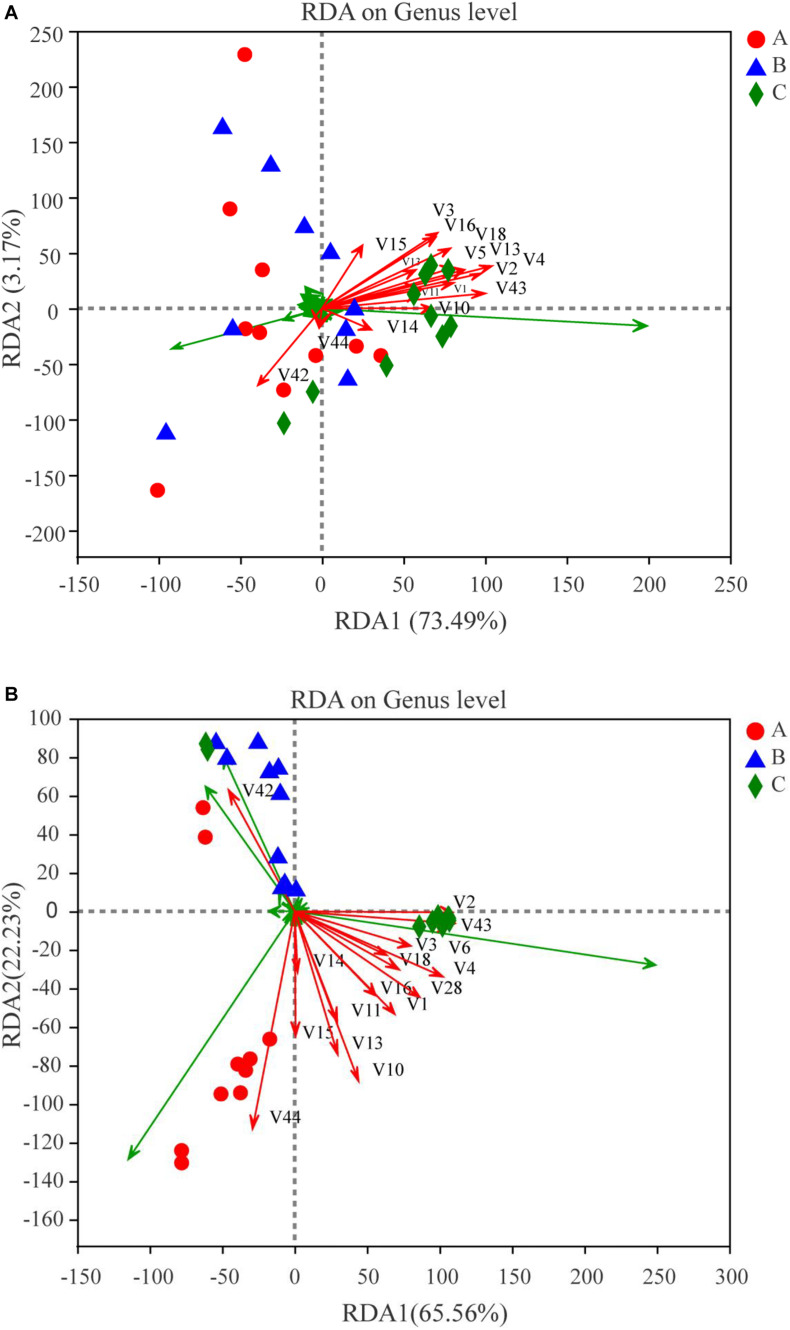
Redundancy analysis (RDA) of dominant microbes (symbols) at levels of genus and major flavor compounds (arrows). The values of axes 1 and 2 are the percentages explained by the corresponding axis. Red dots, blue triangular and green diamond represent Group A, B and C, respectively. **(A)** For prokaryotic microbiota; **(B)** for eukaryotic microbiota.

**FIGURE 9:**
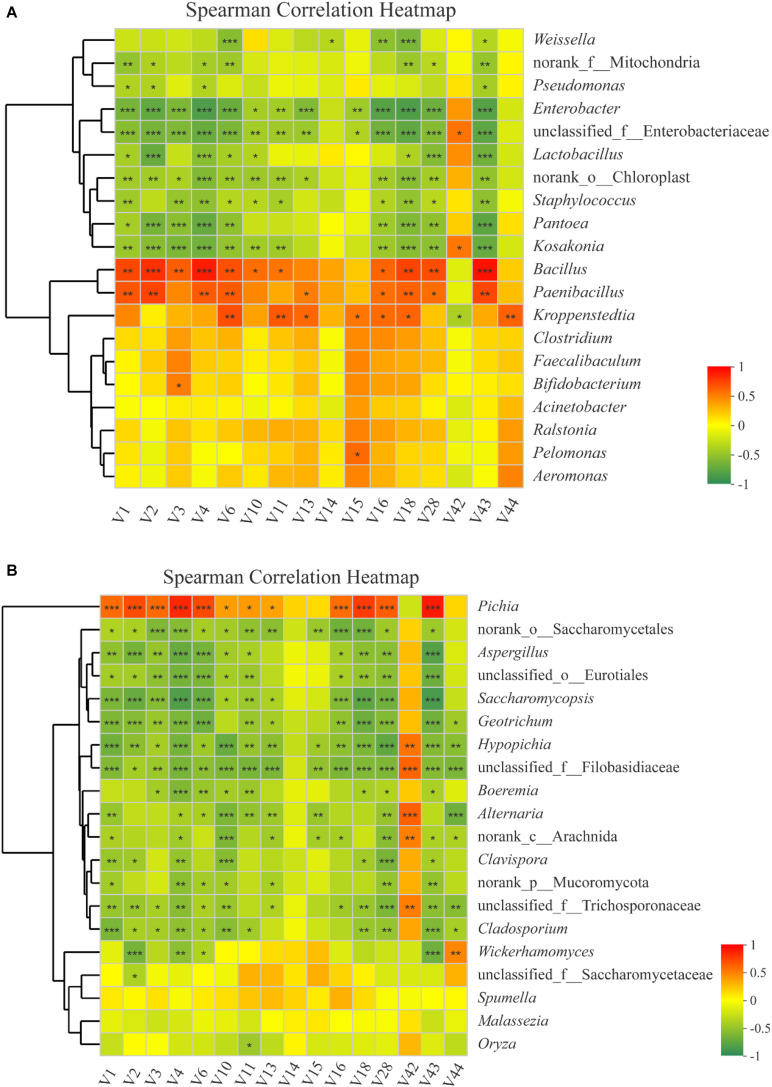
Correlation between partial flavor compounds (V1–V44: the codes of different flavor compounds in Supplementary Table S1) and prokaryotic microbiota **(A)** and eukaryotic microbiota **(B)**. Scale bar colors denote the nature of the correlation, with 1 indicating a perfectly positive correlation (red) and –1 indicating a perfectly negative correlation (green) between flavor compounds and prokaryotic microbiota or eukaryotic microbiota. Asterisk shows significant correlations (“*”, 0.01 < *P* < 0.05; “**”, 0.001 < *P* < 0.01; “***”, *P* < 0.001), and NA shows no correlations.

In this study, RDA was conducted to reveal the possible relationships between microbial community composition and flavor compounds (Figure 8A). Overall, the two axes explained 76.66% of the variation in prokaryotic community differentiation, which suggests a strong correlation between the prokaryotic community and flavor compounds. RDA (Figure 8A) revealed that the volatiles could be divided into two groups on the basis of their loading on the axes. Group I contained 14 volatile compounds. Most of these were higher alcohols and esters, which had a positive association with *Bacillus* and *Paenibacillus*. Two compounds, guaiacol and 2-methoxy-4-vinylphenol ethylguaiacol, were categorized into Group II and were strongly positively -correlated with *Pantoea*, *Kosakonia*, and *Enterobacter*. *Bacillus* is an important genus during *Baijiu* production because it can secrete many enzymes such as lipase, amylase, and protease, which can convert protein and starch into esters and other flavor compounds (Fan et al., 2020). A previous report found that *Pantoea* and *Enterobacter* can produce guaiacol (Dillon et al., 2002). Correlation analysis was used to evaluate the relationship between volatile compounds and microbial communities (Figure 9A). The analysis showed a significant correlation between various flavor substances and the microbial community structure. *Bacillus* are positively correlated with the production of higher alcohols and some esters, including ethyl acetate, ethyl butyrate, ethyl octanoate, ethyl caprate, phenethyl acetate, and 4-ethylguaiacol because of the many enzymes produced by this genus (Fan et al., 2020). The correlation of *Paenibacillus* with flavor compounds was similar to that of *Bacillus*, except there was no significant correlation with isobutanol, but there was a significant correlation with ethyl caproate. As reported in previous studies, *Paenibacillus* can produce organic acids, higher alcohols and esterases that can convert organic acid into esters (Wang et al., 2014; Zhang et al., 2017). *Kroppenstedtia* was mainly positively -correlated with the production of esters, such as ethyl butyrate, ethyl caproate, ethyl heptanoate, ethyl octanoate, and ethyl caprate. In addition, *Kroppenstedtia* were positively -correlated with β-phenylethanol and 2-methoxy-4-vinylphenol ethylguaiacol. *Kroppenstedtia* was recently identified in *Baijiu* brewing, and knowledge about its functional characteristics is limited (Fan et al., 2020). Other members of the prokaryotic community were negatively correlated with flavor compounds. *Weissella* was negatively -correlated with β-phenylethanol, ethyl lactate, ethyl octanoate, ethyl caprate, and 4-ethylguaiacol. Some previous studies showed that *Weissella* was positively connected with ethyl lactate and ethyl ester (Wang et al., 2018; Xu et al., 2018). This difference in results may be due to the proportion of *Weissella* in the microbial communities and its relationship with other members of the microbial communities. Negative correlations were also found between *Pseudomonas* and higher alcohols and 2-methoxy-4-vinylphenol ethylguaiacol. Except for ethyl lactate, guaiacol and 2-methoxy-4-vinylphenol ethylguaiacol, there were significant negative correlations between *Enterobacter* and flavor compounds. As previously reported, although the role of *Enterobacter* in *Baijiu* production remains unclear, it has been found to produce lipase, which contributes to the formation of esters (Fan et al., 2020). *Lactobacillus* was negatively -correlated with higher alcohols, ethyl acetate, ethyl caprate, phenethyl acetate, and 4-ethylguaiacol, which was inconsistent with previous reports (Pang et al., 2018). The reason for this discrepancy may be similar to that suggested above for *Weissella*. *Staphylococcus* was also negatively -correlated with higher alcohols, ethyl acetate, ethyl butyrate, ethyl octanoate, ethyl caprate, phenethyl acetate, and 4-ethylguaiacol, which is inconsistent with previous reports and may be caused by lipase produced by *Staphylococcus* (Horchani et al., 2012; Tang et al., 2019). In addition to higher alcohols, *Pantoea*, whose function in *Baijiu* production remains unknown, was significantly negatively correlated with ethyl octanoate, ethyl caprate, phenethyl acetate, and 4-ethylguaiacol (Fan et al., 2019a). There was also a negative correlation between *Kosakonia* and these flavor compounds, which was a positive correlation with *Bacillus*.

**FIGURE 10:**
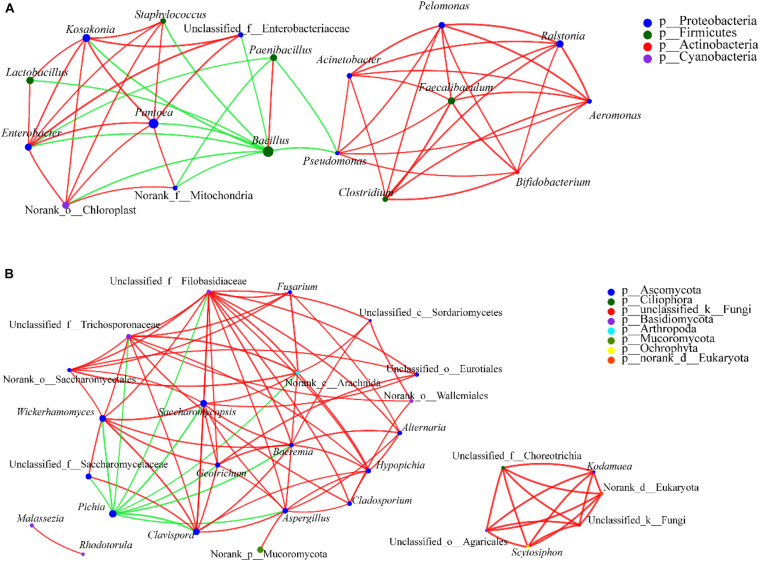
Correlation network of co-occurring genera in dominant microbiota. Statistical significance (*P* < 0.05) and Spearman correlation coefficient (| ρ| < 0.5) indicate the correlations. Green and red lines indicate negative and positive interactions, respectively, between genera. The thickness of the lines represents the strength of interaction. Size of each node is proportional to the number of connections, the nodes with the same color can be affiliated to the same phylum. The thickness of edge is proportional to the absolute value of Spearman’s correlation coefficients. **(A)** For prokaryotic microbiota and **(B)** for eukaryotic microbiota.

To predict the ecological relationships across different bacterial communities, spatial Pearson’s correlations between bacterial species were visualized and then analyzed (Figure 10A). In the network, pairwise relationships were represented by edges connecting two nodes. At the genus level, there were 544 associations among 46 nodes in the control (Group C), 412 associations among 48 nodes in Group A, and 454 associations among 48 nodes in Group B, which indicated decreased network complexity in the latter groups. Conversely, after bioaugmentation with *Bacillus*, the complexity of interspecies interactions in microecosystems increased (He et al., 2019a, 2020). These different interaction effects may be due to the use of different microorganisms for bioaugmentation. In addition, the top 10 densest clusters in three groups were different. In other words, the network structure and interrelation of prokaryotic microbiota was changed by adding *W. anomalus*, which may be the reason for the change of *Baijiu* flavor components (Song et al., 2019; He et al., 2019a, 2020). Notably, most genera with the densest clusters were not the genera with the highest richness, that is, the genus may not be a key genus to directly produce flavor substances in *Baijiu*, but is a key genus for maintenance of the balance of the microbial community in *Baijiu* brewing system. This may be why it is difficult for different flavors of *Baijiu* to be successfully simulated by mixed cultivation of dominant genera. However, as He et al. (2020) reported, bioturbation of fortified *Daqu* is feasible for flavor metabolism by interspecies interactions of functional microbiota in *Baijiu* fermentation. This result is worth further verification and may become another direction for understanding *Baijiu* brewing, that is, to study the genera, which are closely related to other genera, especially the dominant genera, in *Baijiu* brewing, and the relationships among these genera. It should be noted that *Bacillus*, *Paenibacillus*, and *Brevibacillus* inhibit the growth of other genera, while they play a synergistic role with each other. The report by He et al. (2020) showed that *Bacillus* was positively -correlated with *Amycolatopsis*, *Mesorhizobium*, and *Methylobacterium*, and negatively -correlated with *Lactobacillus*. The functions of the relationships among these genera in *Baijiu* brewing remain unclear and should be investigated.

### Eukaryotic Community Structure and Diversity

A total of 1,422,210 quality-filtered and chimera-checked 18S rRNA gene sequences were obtained with an average length of 400 bp across all samples. Rarefaction analysis indicated that all eukaryotic communities were well-represented, as rarefaction curves approached saturation (Supplementary Figure S2b). A total of 44 OTUs that belonged to 35 genera and 11 phyla were identified using a 97%-identity 18S rRNA sequence cutoff. The change of species richness with fermentation stage was similar in Groups A and B, but different in Group C. The richness in Groups A and B decreased in the first half of the fermentation (days 0–12) and remained stable in the second half of the fermentation (days 12–24); in Group C, it decreased and slightly increased in the first and second halves of the fermentation, respectively (Table 1). The diversity was significantly different in each group by Wilcoxon rank-sum test of the Shannon and Simpson indexes (Supplementary Figures 3c,d). The lowest diversity was observed in Group C, and the highest diversity in Group B. That is, the eukaryotic diversity increased after addition of *W. anomalus* to the SSF. In Group A, the diversity decreased in the early stage of the fermentation (days 0–6), then increased and stabilized in the middle, and final stages. In Group B, the diversity decreased in the first half of the fermentation (days 0–12), then increased then stabilized in the second half. In Group C, the diversity decreased throughout the process (Table 1). In general, the eukaryotic community structure changed in the same way as the prokaryotic community during SSF after addition of *W. anomalus*.

Eleven phyla were observed in the eukaryotic communities: Basidiomycota, Phragmoplastophyta, Ascomycota, Porifera, Mucoromycota, Cercozoa, Ochrophyta, Arthropoda, Ciliophora, norank_d__Eukaryota, and unclassified_k__Fungi. Each group had nine phyla. The most abundant phylum was Ascomycota, with a relative abundance of 90.62–99.61% in the samples, followed by Mucoromycota (0.39–9.19%) (Figures 3C,D). In general, the abundance of Ascomycota was highest in Group C and lowest in Group B, while the opposite was observed for Mucoromycota.

Among the 35 genera detected in these samples, 25 genera were found in each of two groups, and 24 in all three groups (Supplementary Figure S4b). The number of eukaryotic genera detected in Groups A and B was similar (27 and 28, respectively); 31 genera were found in Group C, but the difference in the number of genera among groups was not significant, which was consistent with the Ace and Chao1 indexes. The dominant genera in the three groups were similar, including *Pichia*, *Wickerhamomyces*, Clavispora-Candida_clade, *Saccharomycopsis*, unclassified_f_Saccharomycetaceae, and norank_p_Mucoromycota, which was consistent with other reports (Li et al., 2018). However, the proportions of the dominant genera were different in different groups. In group A, the dominant genus was *Wickerhamomyces*, which accounted for 54.32% abundance, followed by *Pichia* (19.26%), *Saccharomycopsis* (10.78%), Clavispora-Candida_clade (6.72%), unclassified_f_Saccharomycetaceae (5.42%), and norank_p_Mucoromycota (2.73%); in Group B, Clavispora-Candida_clade was the most abundant (31.76%), followed by *Pichia* (26.65%), *Wickerhamomyces* (15.17%), unclassified_f_Saccharomycetaceae (11.36%), *Saccharomycopsis* (10.08%), and norank_p_Mucoromycota (4.37%); while in Group C, the eukaryotic community was dominated by *Pichia* (75.77%), followed by *Saccharomycopsis* (9.71%), Clavispora-Candida_clade (6.05%), *Wickerhamomyces* (5.91%), and norank_p_Mucoromycota (1.86%) (Figures 4C,D). This may be one reason for the different presence/abundance of flavor compounds in the different groups, especially esters and higher alcohols. During the SSF process, there were some differences in the changes in genera among groups. In detail, although *Pichia* showed an increasing trend in each group, the amplitude and rate of increase differed; the amplitude of increase was smallest and the rate was lowest in Group A and highest in Group C. In Group A, the amount of *Wickerhamomyces* increased in the early stage of the fermentation (days 0–6), but subsequently decreased; in Group B, the trend was opposite to that in Group A; in Group C, the amount decreased throughout the process. In the whole SSF process, the abundance of *Wickerhamomyces* was highest in Group A and lowest in C, which may be an important reason for the increase in the content of esters, especially the FMEEs in Group A, during fermentation. The abundance of Clavispora-Candida_clade decreased in Groups A and C during the fermentation, and the abundance was similar in Groups A and C throughout the entire process. In Group B, the abundance of Clavispora-Candida_clade increased in the first half of the process then decreased in the second half, and it was higher in Group B than in Group A or C, which may be one reason for the difference of flavor compounds, especially esters, between Groups A and B. The abundance of *Saccharomycopsis* and *Aspergillus* showed a decreasing trend in all three groups, and the amount was similar, especially in Group A and B.

PCoA clearly grouped the fungal communities (Supplementary Figure S6b). Different fermentation samples from the same experimental group clustered, and samples from different experimental groups showed obvious differences. As other studies have shown, after bioaugmentation, the microbial community structure changed (Song et al., 2019; He et al., 2019a, b, 2020). Significant change in the eukaryotic communities was observed after addition of *W. anomalus*. As in PCoA, PLS-DA showed that the eukaryotic communities in different experimental samples clustered separately, which suggests that the overall structures of the eukaryotic communities in the different groups were significantly different (Figure 7B). Spots in Groups A or B showed more dispersed distribution patterns than those for the controls (as was also observed for the prokaryotic communities), which indicates that the microbial community structure was changed by the addition of *W. anomalus*. Enterotype analysis provided a clear visualization of the relationships among the different sample groups (Supplementary Figure S7b). Most of the samples in Group A belonged to the *Wickerhamomyces* enterotype, and a few belonged to the *Saccharomycopsis* enterotype; samples in Group B mainly belonged to the Clavispora-Candida_clade and *Pichia* enterotype and a few belonged to the *Saccharomycopsis* enterotype; while most samples in Group C belonged to the *Pichia* enterotype and a few belonged to the *Saccharomycopsis* enterotype. Therefore, there are great differences among samples from the three groups. Thus, the *W. anomalus* enterotype was only observed for Group A, which may be one reason for the higher ester content in Group A.

**FIGURE 11:**
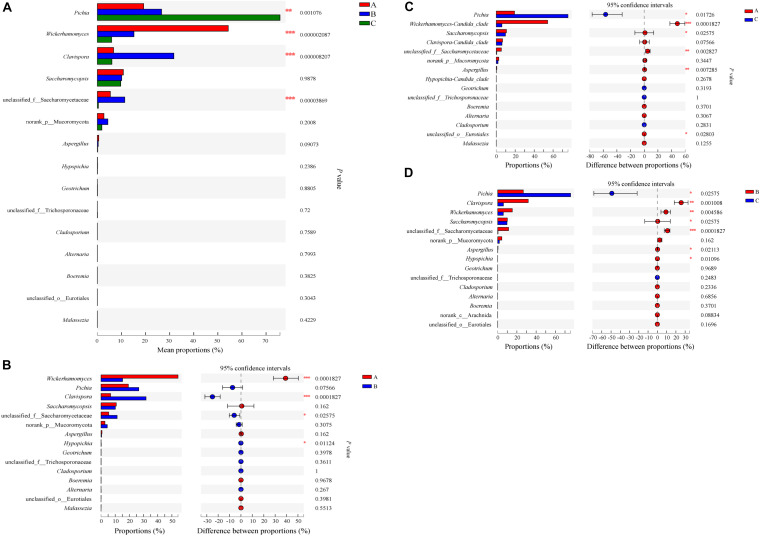
Relative abundances of eukaryotic microbiota that showed significant differences among three groups at genus level. Data of groups were showed as relative abundance of genus in each group. **(A)** A one-way ANOVA was used to evaluate the significance of differences among three groups; **(B–D)** showed that phylotypes significantly different between Groups A and B, Groups A and C, and Groups B and C, respectively, at genus level, and statistical analysis was performed by the Wilcoxon rank-sum test. Asterisk shows significant differences (“*”, 0.01 < *P* < 0.05; “**”, 0.001 < *P* < 0.01; “***”, *P* < 0.001).

We further compared the relative abundances of the eukaryotic communities among the groups at the phylum and genus levels. All phyla were observed in all groups (Supplementary Figure S8). Arthropoda were increased (*P* < 0.05) in Group B compared with Group A. At the genus level, *Pichia*, *Wickerhamomyces*, Clavispora-Candida_clade and unclassified_f_Saccharomycetaceae were statistically different (*P* < 0.01) (Figure 11). When comparing Groups A and B, *Wickerhamomyces* increased in Group A, while Clavispora-Candida_clade, unclassified_f_Saccharomycetaceae, and Hypopichia-Candida_clade increased in Group B. *Pichia* decreased, but *Wickerhamomyces*, *Saccharomycopsis*, unclassified_f_Saccharomycetaceae, *Aspergillus*, and unclassified_o_Eurotiales increased in Group A compared with Group C. Compared with Group C, *Pichia* decreased, and Clavispora-Candida_clade, *Wickerhamomyces*, *Saccharomycopsis*, unclassified_f_Saccharomycetaceae, *Aspergillus*, and Hypopichia-Candida_clade increased in Group B. To identify the distinguishing taxa within the groups, the LEfSe method was implemented (Figures 6C,D). The greatest differences were in the phylum Ascomycota, class Saccharomycetes, and order Saccharomycetales. Phaffomycetaceae (the family to genus) exhibited significantly higher abundance in Group A than in the other groups; Metschnikowiaceae and Saccharomycetaceae (the family to genus) were remarkably prevalent in Group B; and Pichiaceae (the family to genus) were significantly increased in control samples (Group C). The relationship between eukaryotic communities and flavor compounds was also investigated by RDA. As shown in Figure 8B, short-medium-chain fatty acid ethyl esters, including ethyl acetate, ethyl butyrate, ethyl caproate, ethyl lactate, ethyl heptanoate, and 2-methoxy-4-vinylphenol ethylguaiacol were strongly positively -correlated with *Wickerhamomyces* in Group A, which was consistent with a previous report (Fan et al., 2018b). In Group C, aromatic compounds including higher alcohols, ethyl octanoate, ethyl caprate, phenethyl acetate, and 4-ethylguaiacol mainly correlated with the abundance of *Pichia*. In our previous study, we found that *Pichia* was related to increased esterifying activity and to the formation of esters (Fan et al., 2019a). Guaiacol was strongly positively correlated with *Saccharomycopsis*, Clavispora-Candida_clade and norank_p_Mucoromycota in Group B as per other reports (Silva et al., 2013; Qi et al., 2014).

To identify specific eukaryotic community members that are potentially associated with certain flavor compounds, Spearman’s correlation analysis was performed for the top 20 genera according to the relative abundance in all samples. In total, 18 genera were significantly correlated with at least one flavor component (Figure 9B). As in RDA, *Pichia* was positively -correlated with higher alcohols, ethyl acetate, ethyl butyrate, ethyl caproate, ethyl octanoate, ethyl caprate, phenethyl acetate, and 4-ethylguaiacol. Norank_o_Saccharomycetales was negatively -correlated with almost all flavor compounds, except ethyl lactate, guaiacol, and 2-methoxy-4-vinylphenol ethylguaiacol. *Aspergillus* and unclassified_o_Eurotiales were similar to Norank_o_Saccharomycetales, except they showed no correlation with ethyl caproate and ethyl heptanoate. *Saccharomycopsis* were also similar to Norank_o_Saccharomycetales except they showed no correlation with ethyl heptanoate. *Geotrichum* were negatively -correlated with higher alcohols, ethyl butyrate, ethyl caproate, ethyl octanoate, ethyl caprate, phenethyl acetate, 4-ethylguaiacol, and 2-methoxy-4-vinylphenol ethylguaiacol. In addition to the flavor compounds associated with *Geotrichum*, Hypopichia-Candida_clade and unclassified_f_Filobasidiaceae had a negative correlation with ethyl acetate. *Boeremia* were negatively -correlated with isobutanol, isoamyl alcohol, β-phenylethanol, ethyl acetate, ethyl butyrate, ethyl caprate, phenethyl acetate, and 4-ethylguaiacol. *Alternaria* were negatively -correlated with isoamyl alcohol, β-phenylethanol, ethyl acetate, ethyl butyrate, ethyl caproate, ethyl heptanoate, phenethyl acetate, and 2-methoxy-4-vinylphenol ethylguaiacol. Norank_c_Arachnida negatively -correlated with isoamyl alcohol, ethyl acetate, ethyl caproate, ethyl heptanoate, ethyl octanoate, phenethyl acetate, 4-ethylguaiacol, and 2-methoxy-4-vinylphenol ethylguaiacol. Clavispora-Candida_clade was negatively -correlated with *n*-hexanol, isoamyl alcohol, ethyl acetate, ethyl caprate, phenethyl acetate, and 4-ethylguaiacol. Norank_p_Mucoromycota was negatively -correlated with isoamyl alcohol, β-phenylethanol, ethyl acetate, ethyl caproate, phenethyl acetate, and 4-ethylguaiacol. unclassified_f_Trichosporonaceae were negatively -correlated with the flavor compounds that correlated with Hypopichia-Candida_clade, except ethyl butyrate and ethyl heptanoate. *Cladosporium* was also similar to Hypopichia-Candida_clade except with respect to ethyl caproate, ethyl heptanoate, and ethyl octanoate. *Wickerhamomyces* were negatively -correlated with most of the higher alcohols and 4-ethylguaiacol. unclassified_f_Saccharomycetaceae were negatively -correlated with *n*-hexanol, and *Oryza* with ethyl butyrate. Guaiacol was positively -correlated with Hypopichia-Candida_clade, unclassified_f_Filobasidiaceae, Alternaria, norank_c-Arachnida, and unclassified_f_Trichosporonaceae. *Wickerhamomyces* was positively -correlated with 2-methoxy-4-vinylphenol ethylguaiacol.

The interactions among eukaryotic communities were explored using co-occurrence patterns and Spearman’s rank correlations (Figure 10B). The results showed that genera from the phylum Ascomycota had a high co-occurrence with genera from the phyla Mucoromycota, Ciliophora, Basidiomycota, Arthropoda, Ochrophyta, Phragmoplastophyta, and so on. Meanwhile, interactions among genera belonging to the Ascomycota were relatively strong. The ethyl acetate-producing genus *Wickerhamomyces* displayed a significant negative correlation with *Pichia* and a positive correlation with *Saccharomycopsis* and *Aspergillus*. *Pichia* (with 11 edges in the analysis), *Saccharomycopsis* (with 12 edges), and *Aspergillus* (with 11 edges) had the most correlations with other genera, and the correlations were different, *Pichia* having negative correlations with all other relevant genera, and *Saccharomycopsis* and *Aspergillus* having positive correlations. *Wickerhamomyces* may, through the interactions with *Pichia*, *Saccharomycopsis*, and *Aspergillus*, regulate other microorganisms in the brew, with effects on the flavor of the *Baijiu*. In addition, it should be noted that, as for the prokaryotic communities, the eukaryotic genera that had the densest clusters were not the genera with the highest richness. Rather, such genera may connect and regulate the microbial communities in the *Baijiu* brewing system. Compared with control samples, the association decreased in experimental groups, which indicates reduced network complexity. In other words, the addition of *W. anomalus* interferes with the microbial community and the relationships between microbial taxa. Some previous reports also showed that bioaugmentation could influence the microbial communities to regulate the properties of *Baijiu* (He et al., 2019a, b, 2020).

## Conclusion

We analyzed the changes of flavor substances in *Baijiu* and the microbial community structure in experimental SSF groups to which two different *W. anomalus* strains were, respectively, added. The flavor of *Baijiu* can be improved by adding *W. anomalus*, but the reasons are complex. The *W. anomalus* play a role themselves. Addition of *W. anomalus* also causes changes in the wider microbial community structure, which is important for improving flavor substances. In conclusion, when using functional microorganisms in *Baijiu* brewing to improve *Baijiu* quality, in addition to the characterization of individual functional microorganisms, it is necessary to analyze how they affect the overall microbial community structure in the brew. Additionally, although the content of esters increased after adding *W. anomalus* Y3604, the content of ethyl alcohol decreased, that is, the yield of *Baijiu* decreased. Therefore, we will next use simultaneous *W. anomalus* Y3604 and *S. cerevisiae* fortification to investigate the effect on the ethanol and ester content.

## Data Availability Statement

The datasets presented in this study can be found in online repositories. The names of the repository/repositories and accession number(s) can be found in the article/Supplementary Material.

## Author Contributions

WW and GF performed the experiments, analyzed the data, and wrote the manuscript. ZF and XL helped to modify the graphs. XL assisted the manuscript checking. BS provided the assistance and guidance throughout the research. All the authors contributed to the article and approved the submitted version.

## Conflict of Interest

The authors declare that the research was conducted in the absence of any commercial or financial relationships that could be construed as a potential conflict of interest.

## Footnotes

1 http://drive5.com/uparse/

2 http://rdp.cme.msu.edu/

3 http://vegan.r-forge.r-project.org/
