# Supplementary material for: Application of Wickerhamomyces anomalus in Simulated Solid-State Fermentation for Baijiu Production: Changes of Microbial Community Structure and Flavor Metabolism
Source: Front Microbiol. 2020 Nov 27;11:598758. doi: 10.3389/fmicb.2020.598758 (PMC7728721; doi:10.3389/fmicb.2020.598758)
Supplement: Supplementary file 1 [file Data_Sheet_1.docx]

**Supplementary material for**

Application of *Wickerhamomyces anomalus* in simulated solid-state fermentation for *Baijiu* production: changes of microbial community structure and flavor metabolism

**Figure legends**

**FIGURE S1** | Flow diagram of simulated solid-state fermentation. The samples were collected at days 0, 6, 12, 18, and 24. Group A， *Daqu* with an initial density of 1 × 10^6^ CFU of *W. anomalus* strain Y3604/mL; Group B, *Daqu* with an initial density of 1 × 10^6^ CFU *W. anomalus* strain YF1503/mL; and Group C (the control group), *Daqu* with 0.9% normal saline solution instead of a strain suspension.

**FIGURE S2** | Rarefaction analysis was performed to evaluate whether further sequencing would likely detect additional taxa, indicated by a plateau. Different colors represent different samples. Sobs represents observed number of species. (a), for prokaryotic microbiota; (b), for eukaryotic microbiota. The samples taken from different experimental groups at different days were labeled as a combination of corresponding groups and days.

**FIGURE S3** | The difference of microbiota diversity as measured by Shannon index (a and c) and Simpson index (b and d) observed in the microbiota of different groups. Error bars are standard error of mean, and *P* values are from Wilcoxon rank-sum t-test. Asterisk shows significant differences (“*”, 0.01 < *P* < 0.05; “**”, 0.001 < *P* < 0.01; “***”, *P* < 0.001). (a) and (b) show the difference of microbiota diversity observed in the prokaryotic microbiota of different groups; (c) and (d) show the difference of microbiota diversity observed in the eukaryotic microbiota of different groups.

**FIGURE S4** | Venn diagrams showed the number of genus unique and shared between Groups A, B and C. The numbers within the diagrams indicate the number of genus shared between groups or unique in different groups. Different circles represent different groups. (a), Venn diagrams for prokaryotic microbiota; (b) Venn diagrams for eukaryotic microbiota.

**FIGURE S5** | Relative abundances of prokaryotic microbiota that showed significant differences among groups at phylum level. Data of groups were showed as relative abundance of phylum in each group. (a), A one-way ANOVA was used to evaluate the significance of differences among three groups; (b), (c) and (d) showed that phylotypes significantly different between Groups A and B, Groups A and C, and Groups B and C, respectively, at phylum level, and statistical analysis was performed by the Wilcoxon rank-sum test. Asterisk shows significant differences (“*”, 0.01 < *P* < 0.05; “**”, 0.001 < *P* < 0.01; “***”, *P* < 0.001). NA, no differences.

**FIGURE S6** | Principal coordinates analysis (PCoA) of the weighted UniFrac distance matrix representing differences in community structure at genus level among three groups. Red dots, blue triangular and green diamond represent Group A, B and C, respectively. (a), for prokaryotic microbiota; (b), for eukaryotic microbiota.

**FIGURE S7** | The enterotype analysis for different groups. Red dots, blue triangular and green diamond represent Group A, B and C, respectively. (a), for prokaryotic microbiota; (b), for eukaryotic microbiota.

**FIGURE S8** | Relative abundances of eukaryotic microbiota that showed significant differences among three groups at phylum level. Data of groups were showed as relative abundance of phylum in each group. (a), A one-way ANOVA was used to evaluate the significance of differences among three groups; (b), (c) and (d) showed that phylotypes significantly different between Groups A and B, Groups A and C, and Groups B and C, respectively, at phylum level, and statistical analysis was performed by the Wilcoxon rank-sum test. Asterisk shows significant differences (“*”, 0.01 < *P* < 0.05; “**”, 0.001 < *P* < 0.01; “***”, *P* < 0.001). NA, no differences.

**
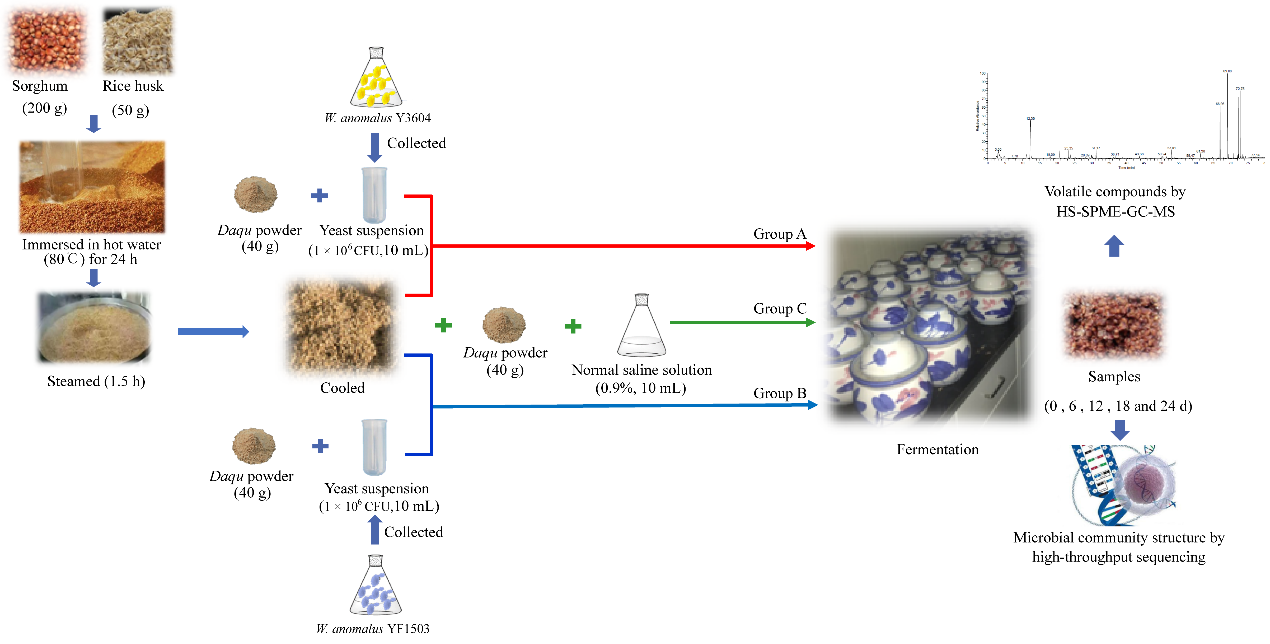
**

**FIGURE S1** | Flow diagram of simulated solid-state fermentation. The samples were collected at days 0, 6, 12, 18, and 24. Group A， *Daqu* with an initial density of 1 × 10^6^ CFU of *W. anomalus* strain Y3604/mL; Group B, *Daqu* with an initial density of 1 × 10^6^ CFU *W. anomalus* strain YF1503/mL; and Group C (the control group), *Daqu* with 0.9% normal saline solution instead of a strain suspension.


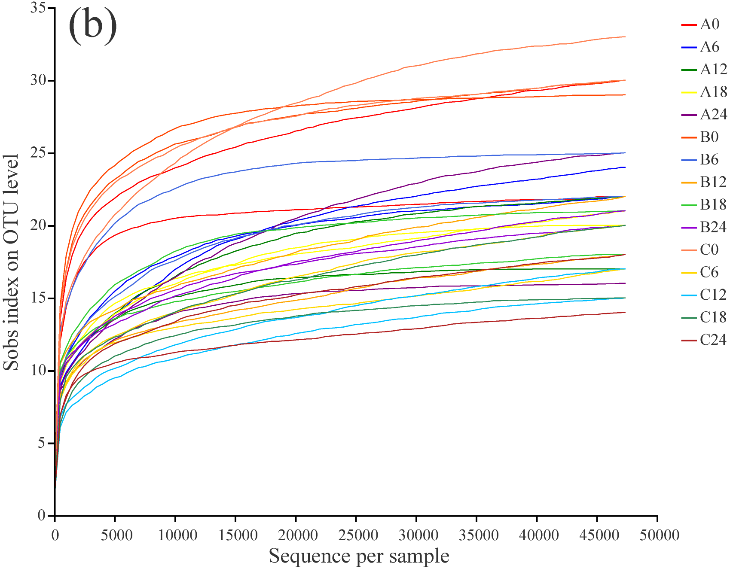

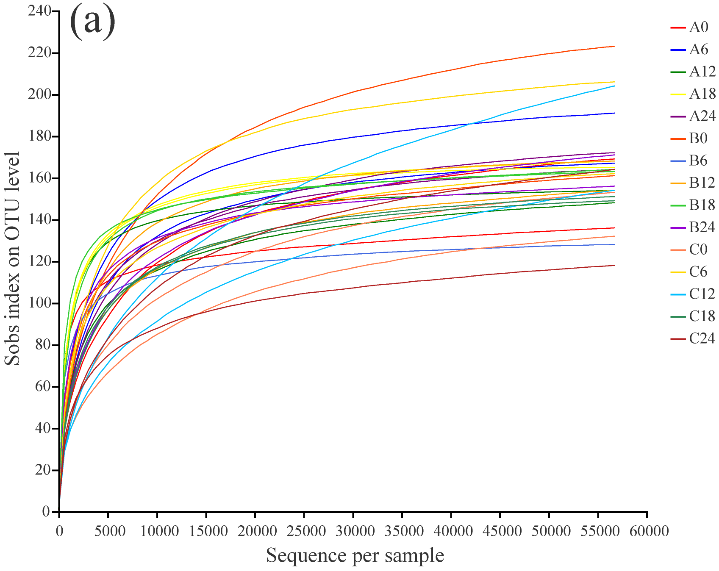


**FIGURE S2** | Rarefaction analysis was performed to evaluate whether further sequencing would likely detect additional taxa, indicated by a plateau. Different colors represent different samples. Sobs represents observed number of species. (a), for prokaryotic microbiota; (b), for eukaryotic microbiota. The samples taken from different experimental groups at different days were labeled as a combination of corresponding groups and days.

**
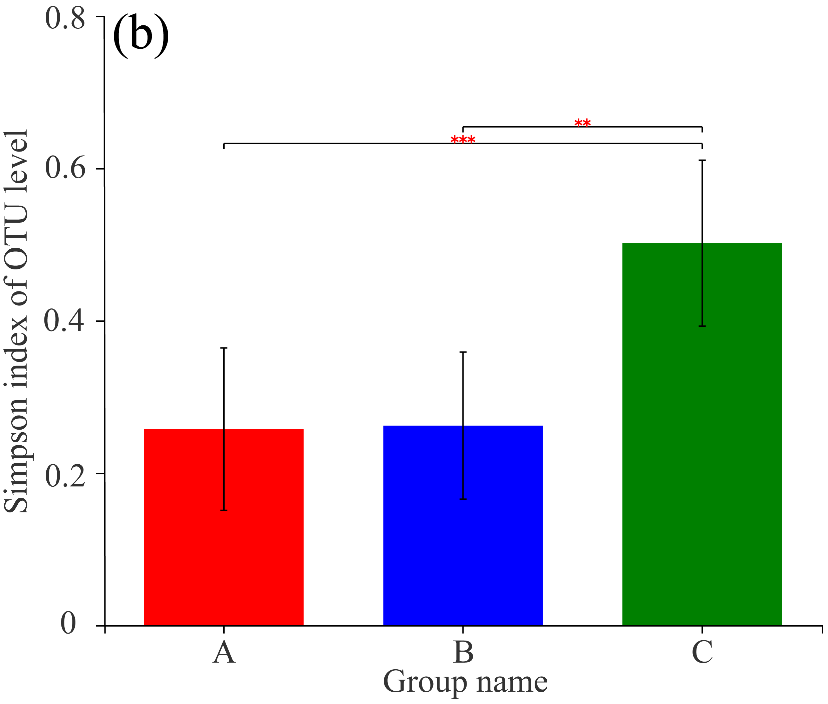

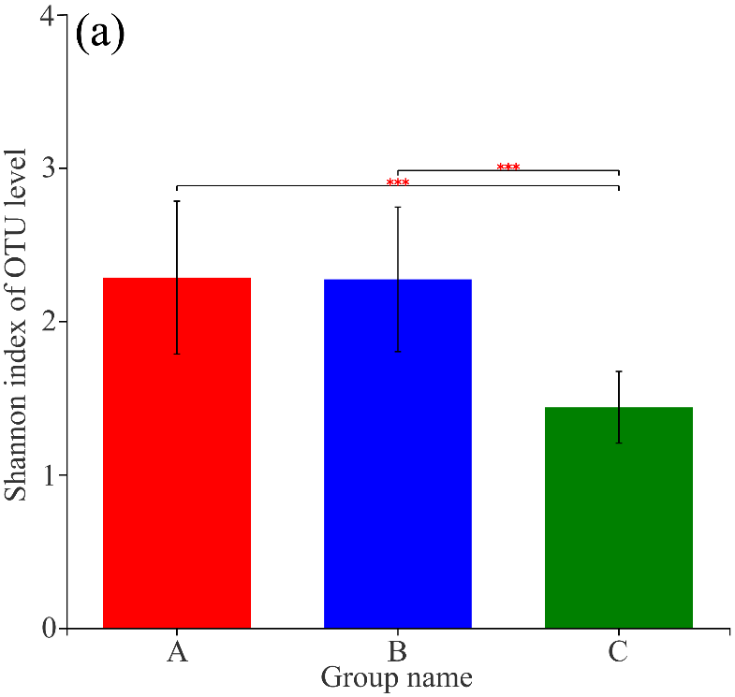
**

**
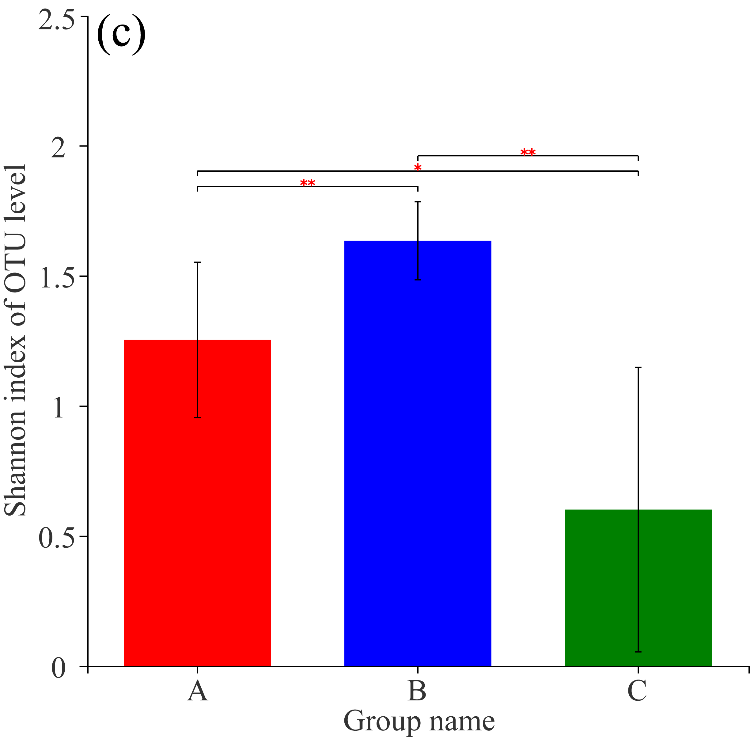
**

**
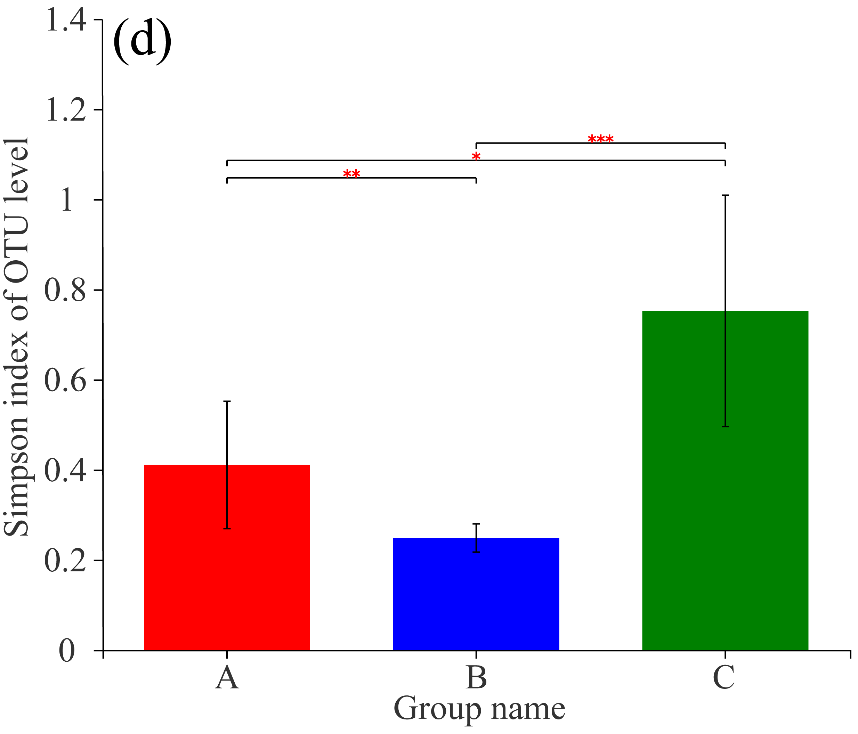
**

**FIGURE S3** | The difference of microbiota diversity as measured by Shannon index (a and c) and Simpson index (b and d) observed in the microbiota of different groups. Error bars are standard error of mean, and *P* values are from Wilcoxon rank-sum t-test. Asterisk shows significant differences (“*”, 0.01 < *P* < 0.05; “**”, 0.001 < *P* < 0.01; “***”, *P* < 0.001). (a) and (b) show the difference of microbiota diversity observed in the prokaryotic microbiota of different groups; (c) and (d) show the difference of microbiota diversity observed in the eukaryotic microbiota of different groups.

**
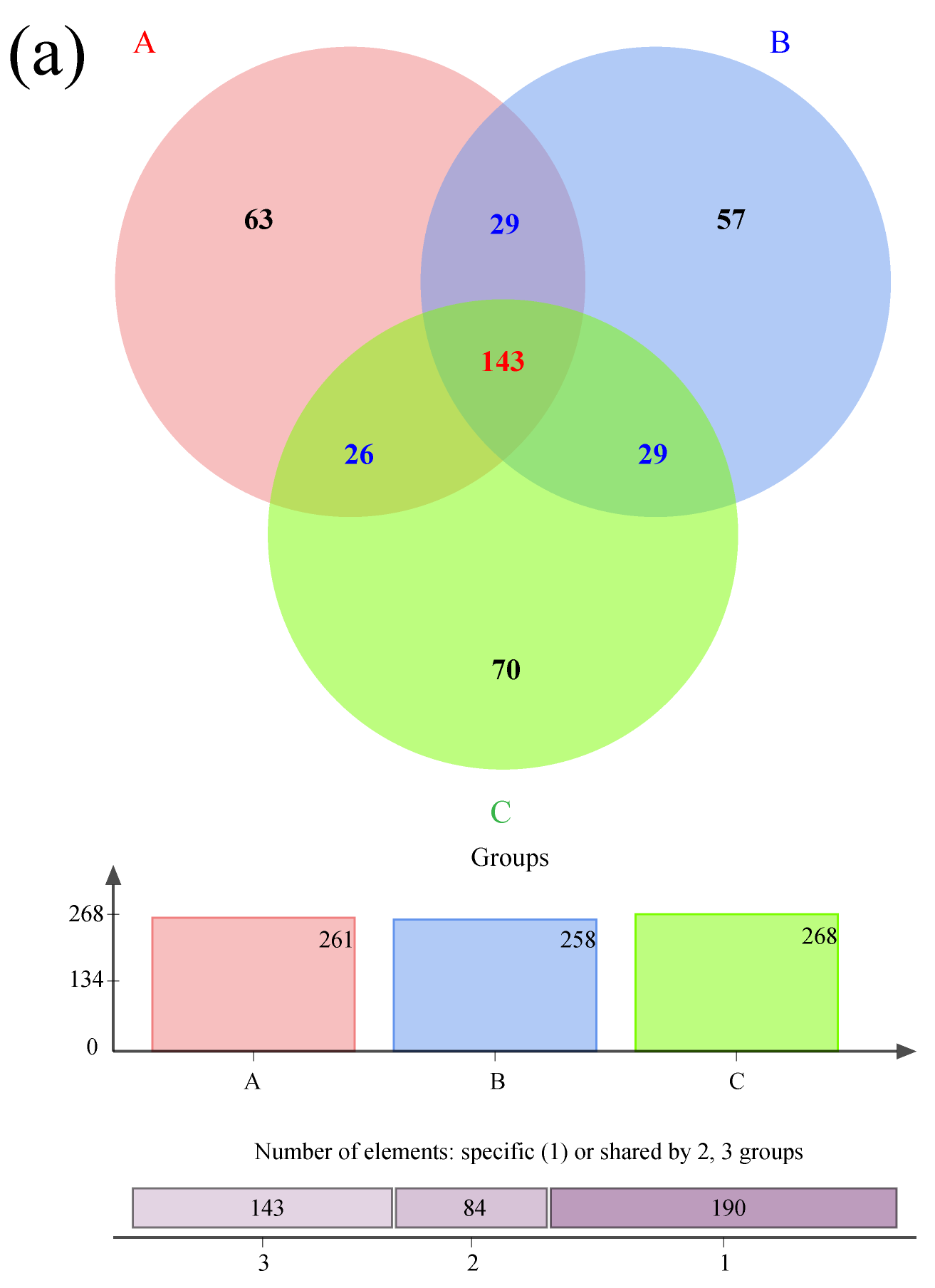
**

**
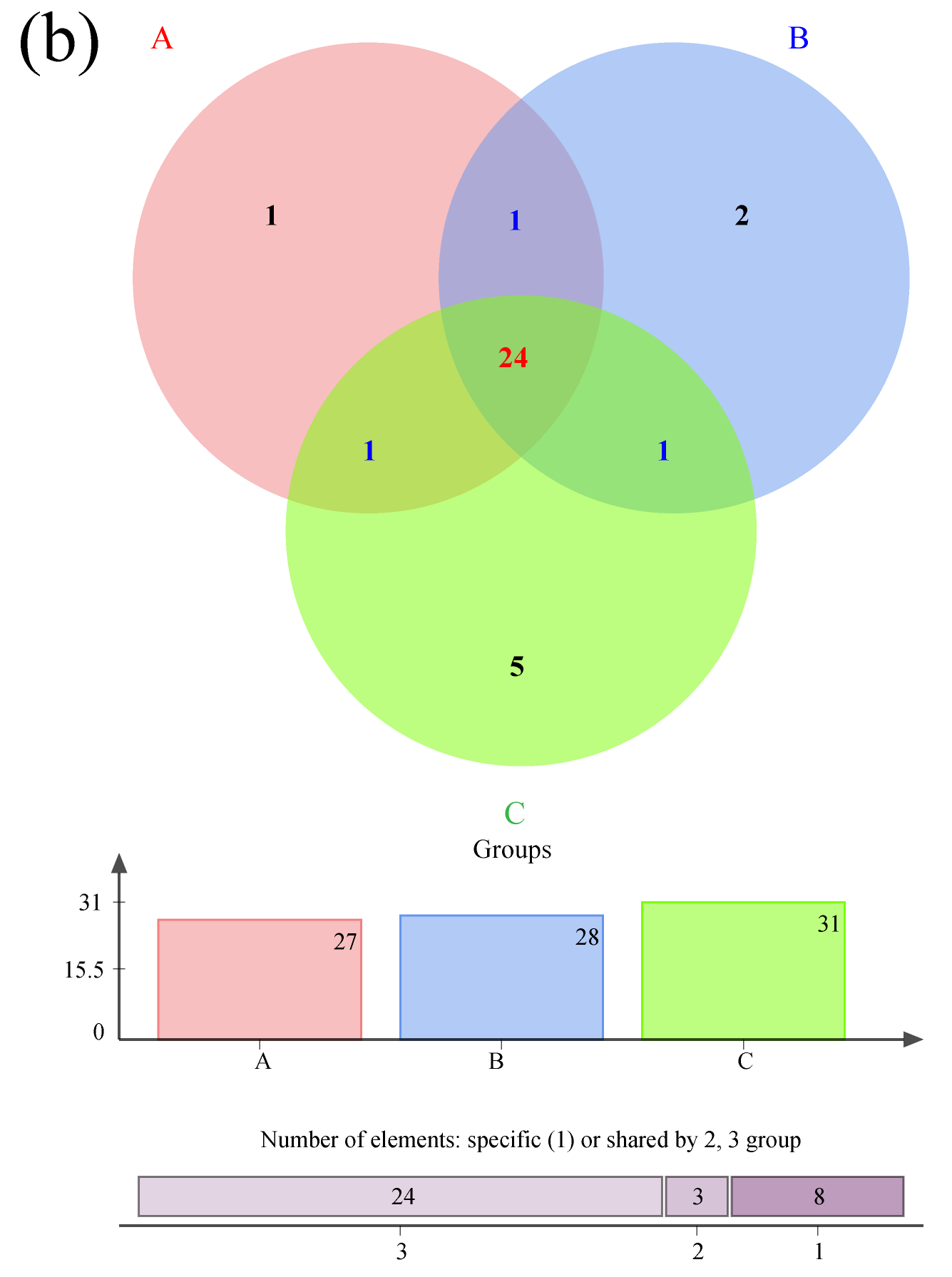
**

**FIGURE S4** | Venn diagrams showed the number of genus unique and shared between Groups A, B and C. The numbers within the diagrams indicate the number of genus shared between groups or unique in different groups. Different circles represent different groups. (a), Venn diagrams for prokaryotic microbiota; (b) Venn diagrams for eukaryotic microbiota.


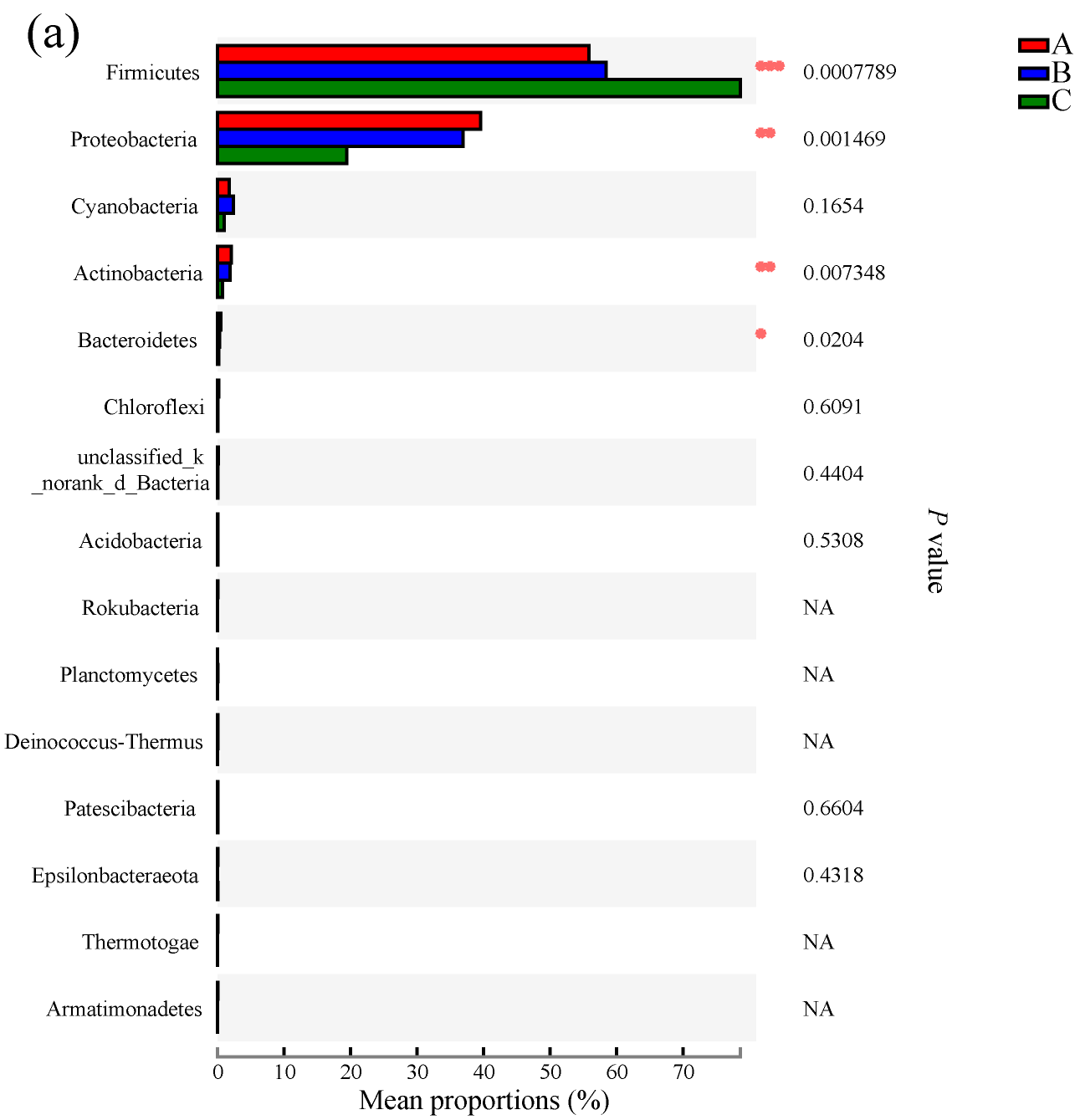


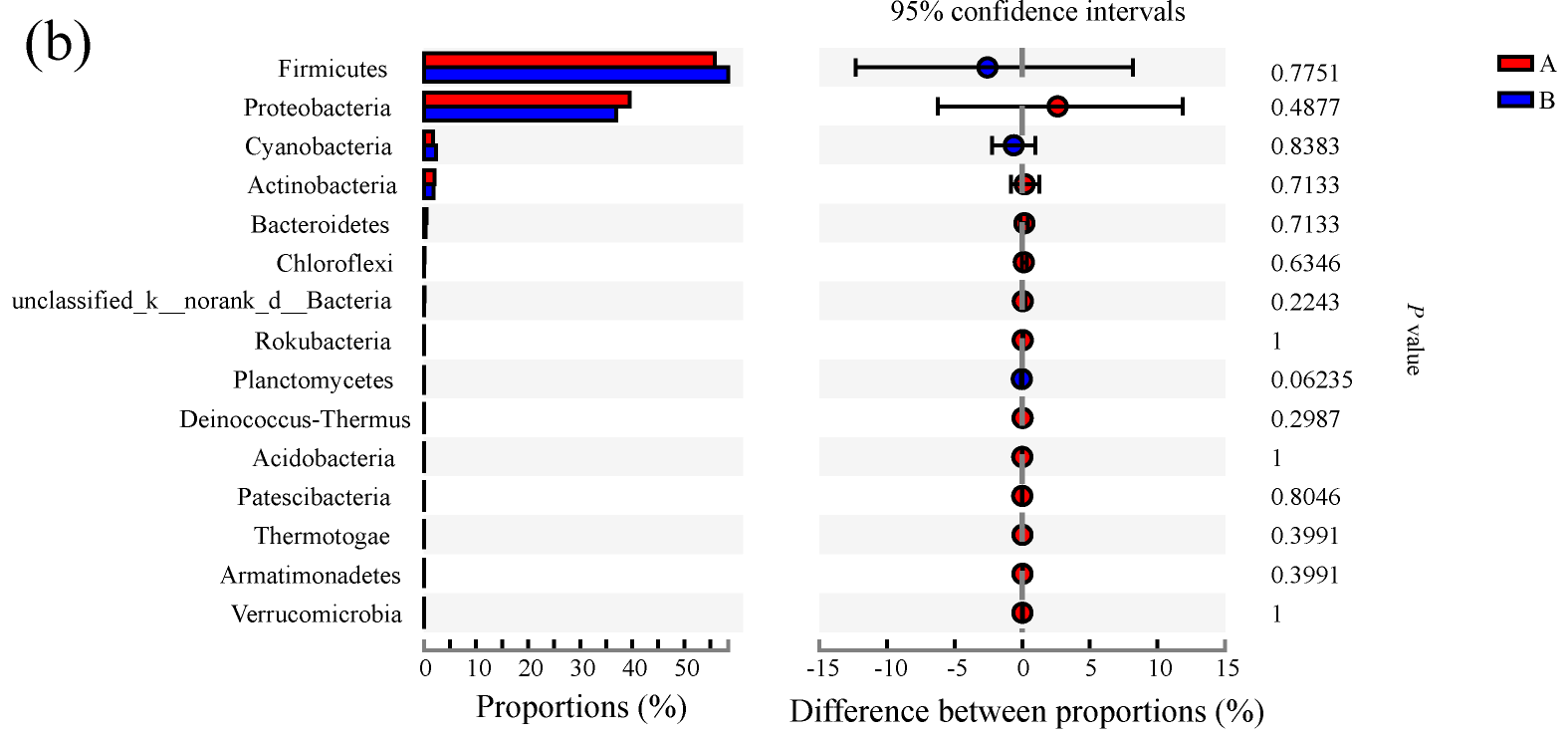


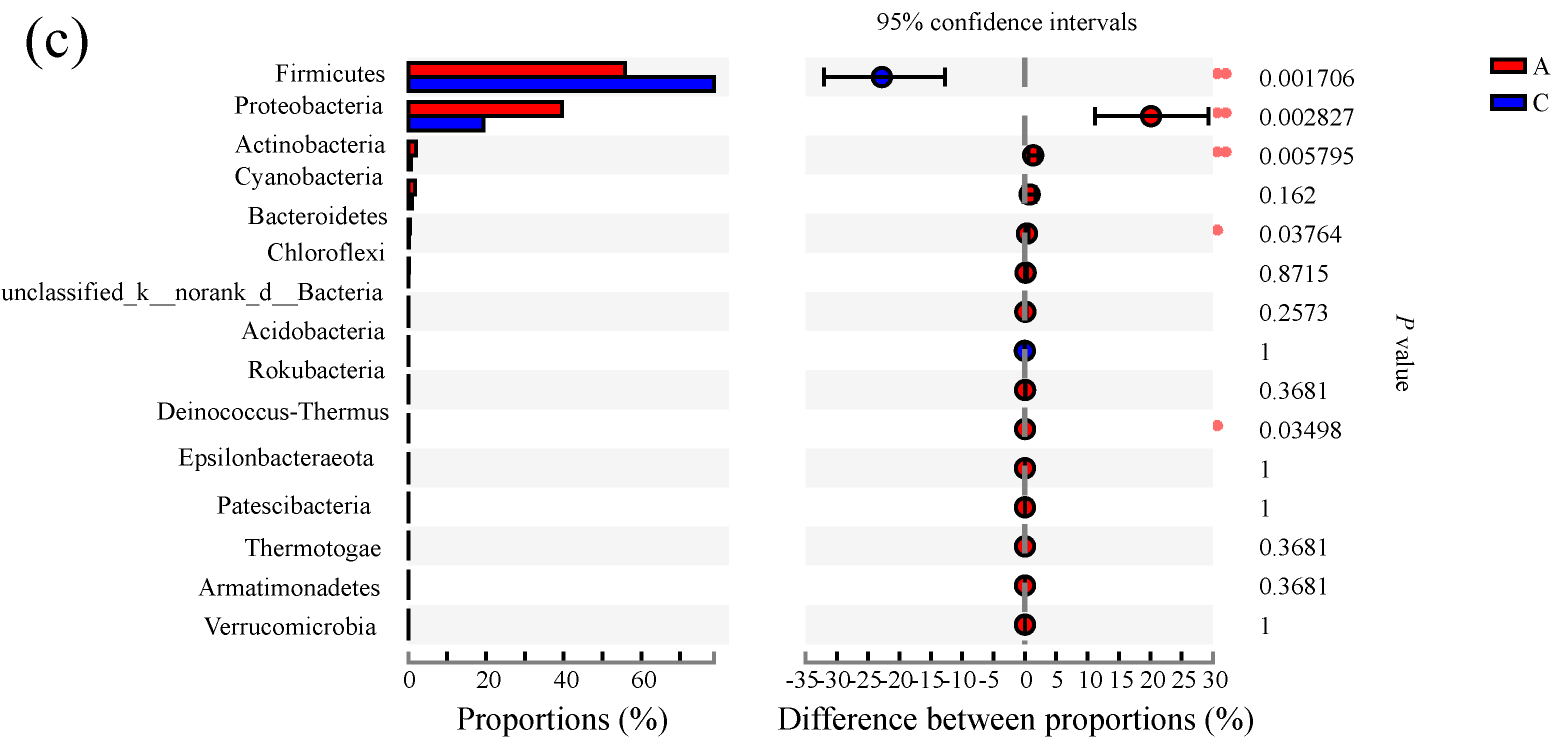


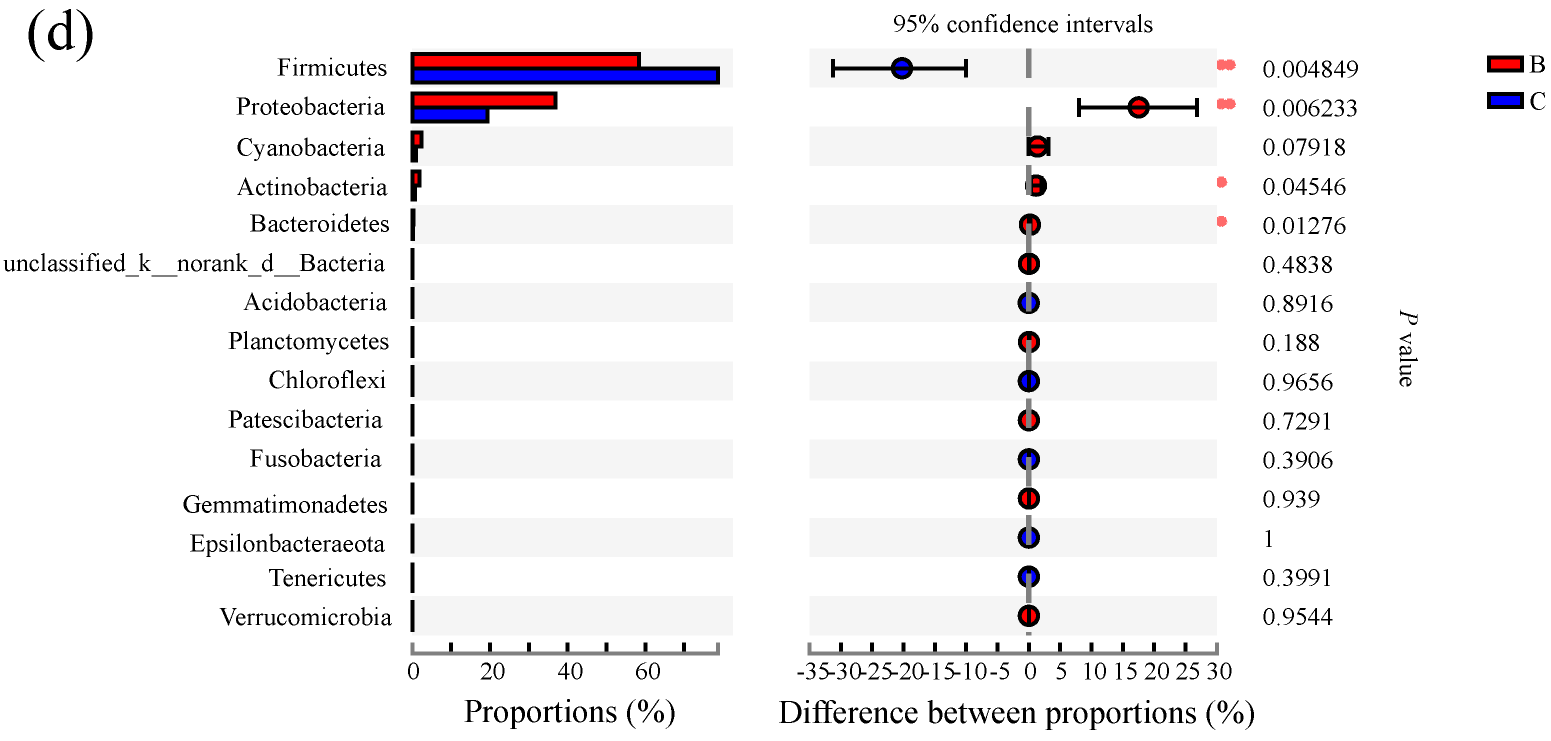


**FIGURE S5** | Relative abundances of prokaryotic microbiota that showed significant differences among groups at phylum level. Data of groups were showed as relative abundance of phylum in each group. (a), A one-way ANOVA was used to evaluate the significance of differences among three groups; (b), (c) and (d) showed that phylotypes significantly different between Groups A and B, Groups A and C, and Groups B and C, respectively, at phylum level, and statistical analysis was performed by the Wilcoxon rank-sum test. Asterisk shows significant differences (“*”, 0.01 < *P* < 0.05; “**”, 0.001 < *P* < 0.01; “***”, *P* < 0.001). NA, no differences.


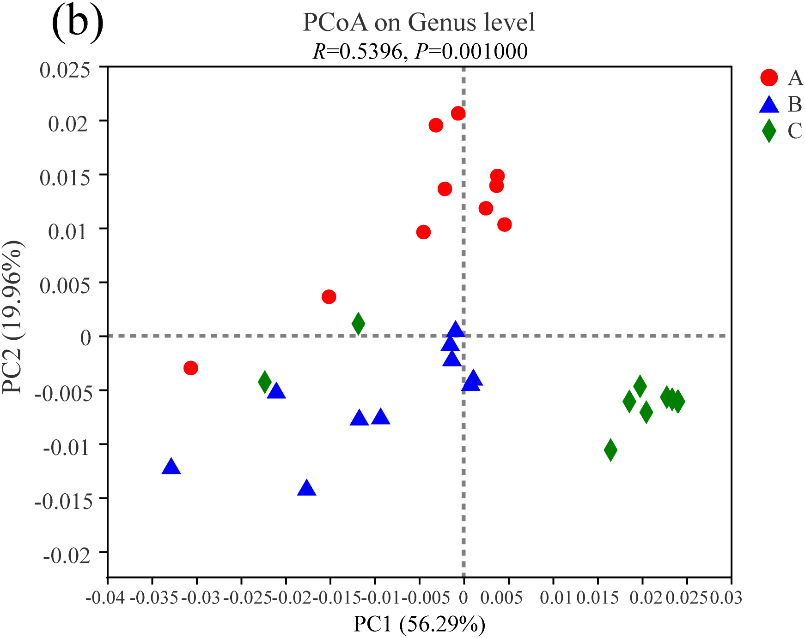

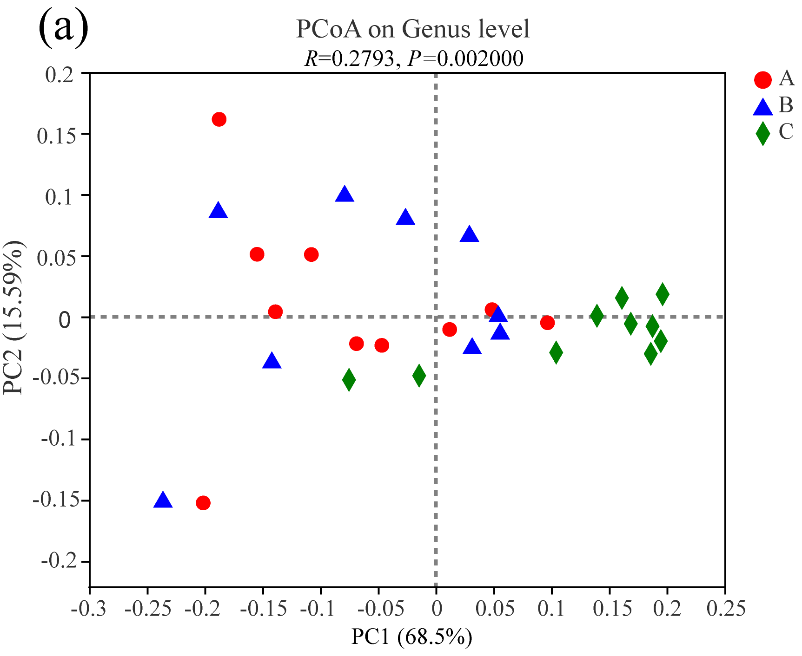


**FIGURE S6** | Principal coordinates analysis (PCoA) of the weighted UniFrac distance matrix representing differences in community structure at genus level among three groups. Red dots, blue triangular and green diamond represent Group A, B and C, respectively. (a), for prokaryotic microbiota; (b), for eukaryotic microbiota.


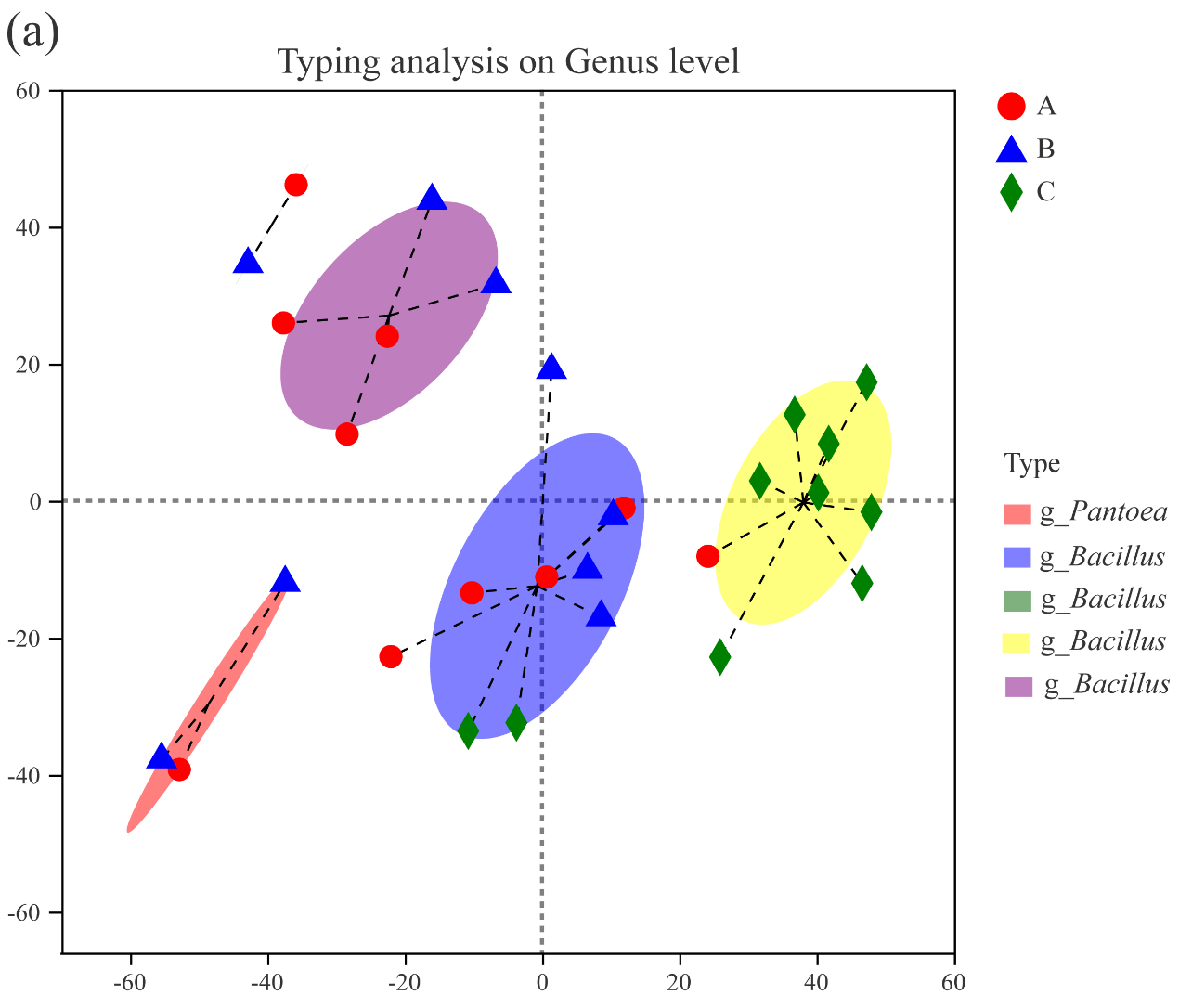


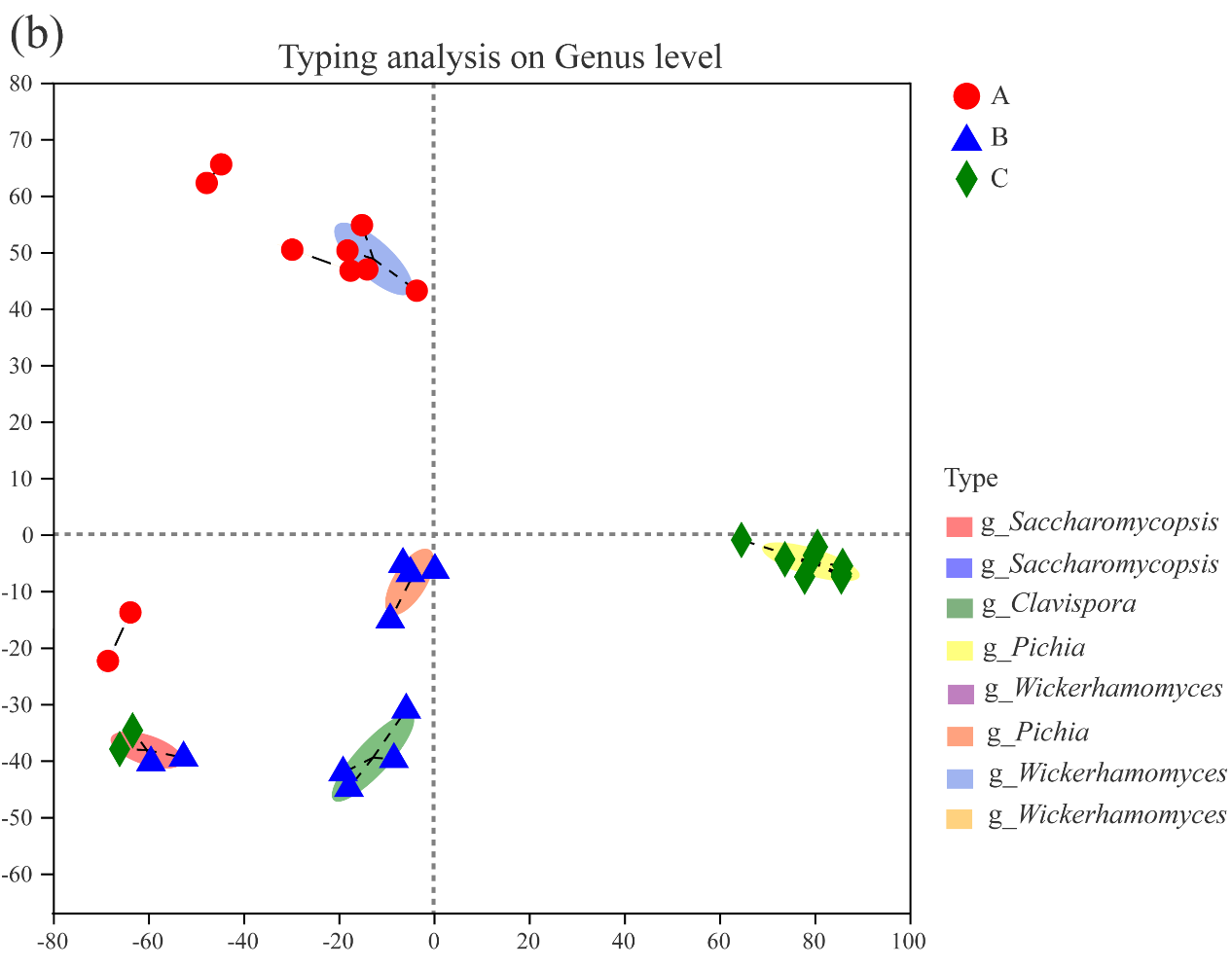


**FIGURE S7** | The enterotype analysis for different groups. Red dots, blue triangular and green diamond represent Group A, B and C, respectively. (a), for prokaryotic microbiota; (b), for eukaryotic microbiota.


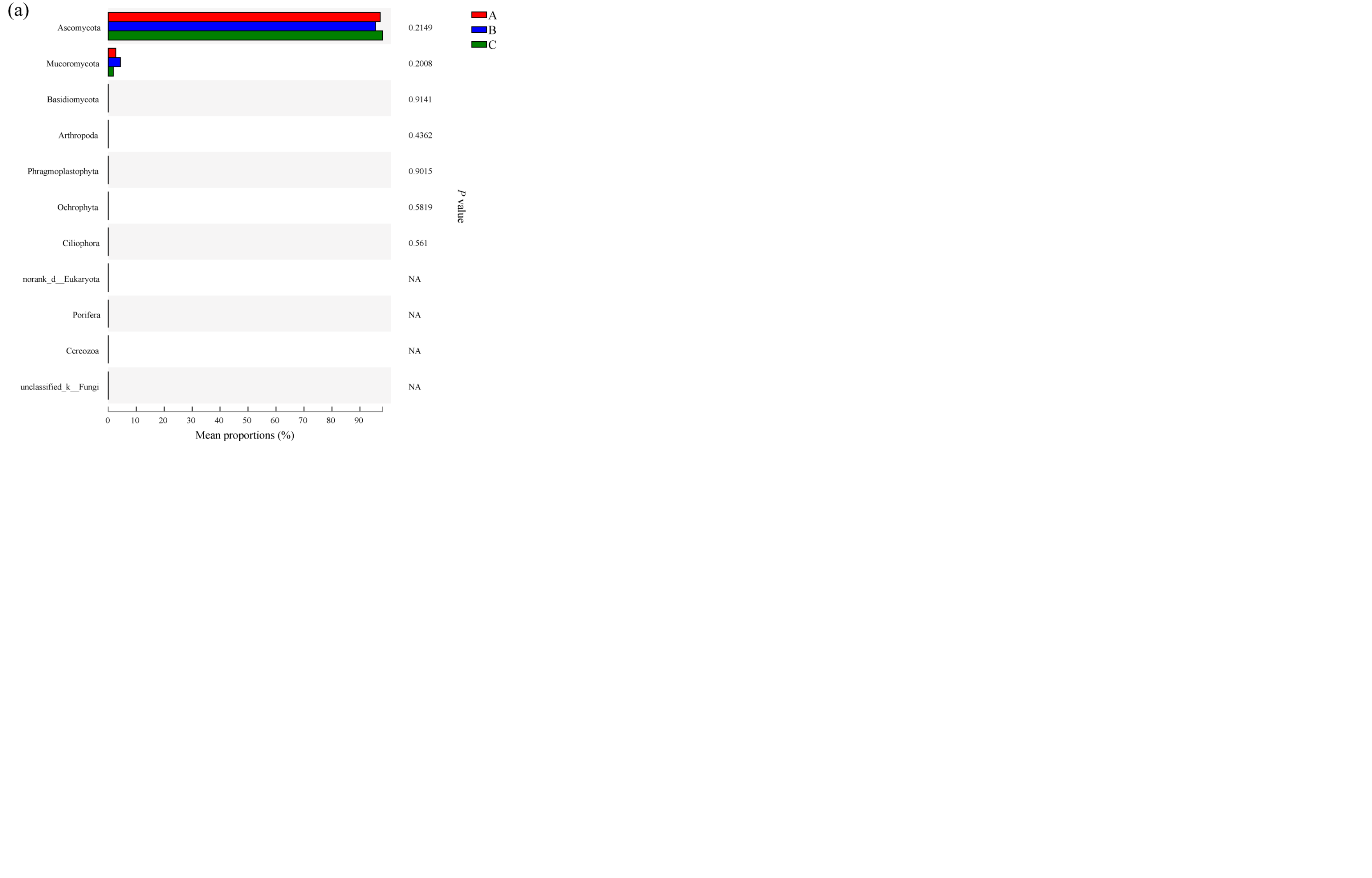


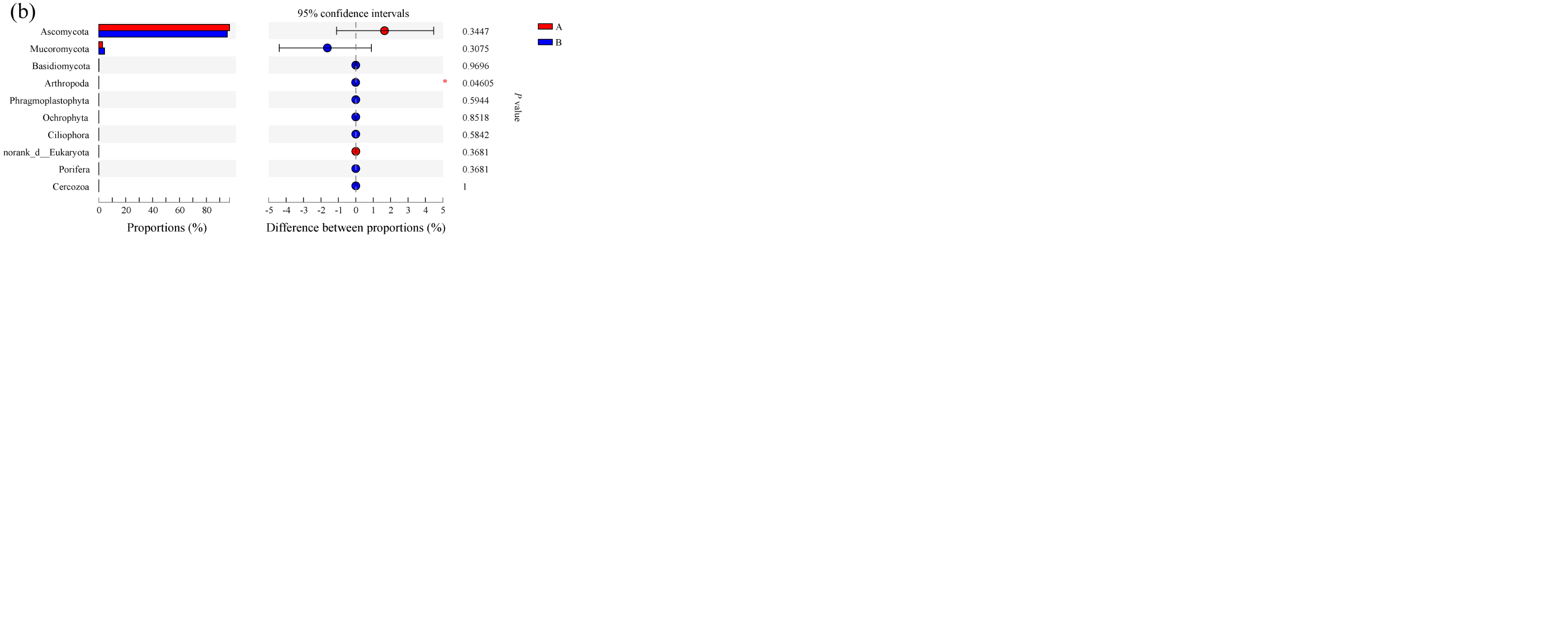


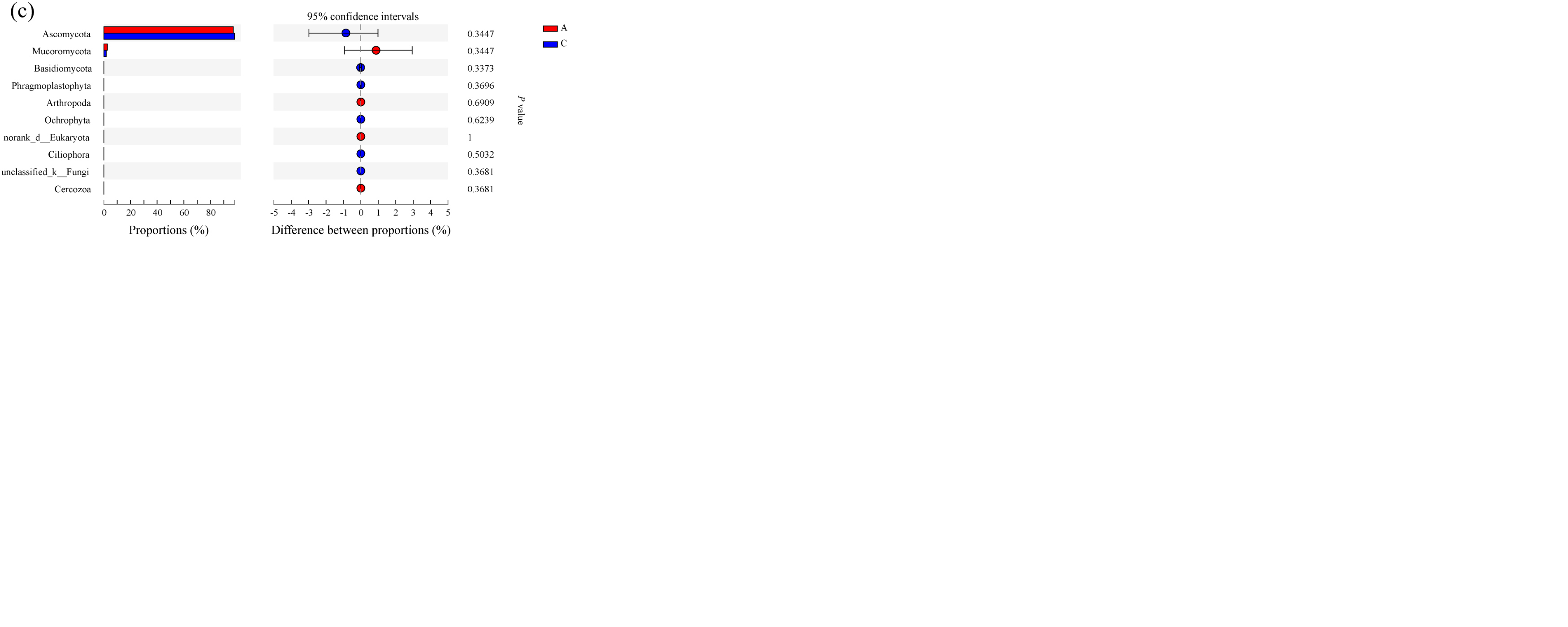


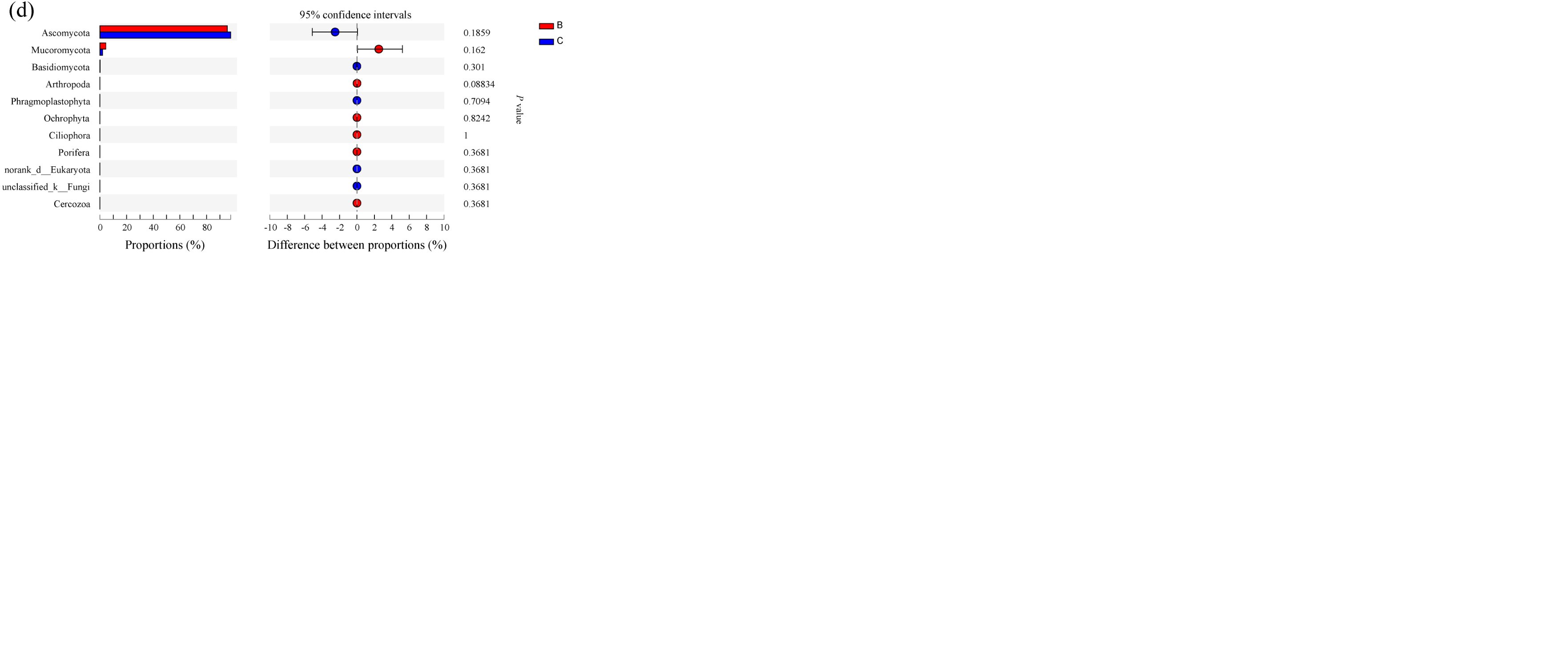


**FIGURE S8** | Relative abundances of eukaryotic microbiota that showed significant differences among three groups at phylum level. Data of groups were showed as relative abundance of phylum in each group. (a), A one-way ANOVA was used to evaluate the significance of differences among three groups; (b), (c) and (d) showed that phylotypes significantly different between Groups A and B, Groups A and C, and Groups B and C, respectively, at phylum level, and statistical analysis was performed by the Wilcoxon rank-sum test. Asterisk shows significant differences (“*”, 0.01 < *P* < 0.05). NA, no differences.

**Supplementary Table S1** | Flavor compounds identified by HS-SPME-GC-MS in samples from the three groups.

| Compounds ID | Compounds (mg/Kg) | Group A | | | | | Group B | | | | | Group C | | | | |
| --- | --- | --- | --- | --- | --- | --- | --- | --- | --- | --- | --- | --- | --- | --- | --- | --- |
|  |  | 0 d | 6 d | 12 d | 18 d | 24 d | 0 d | 6 d | 12 d | 18 d | 24 d | 0 d | 6 d | 12 d | 18 d | 24 d |
| **Alcohols** | | | | | | | | | | | | | | | | |
| V1 | Ethanol | 0.011±0.001 | 2.324±0.202 | 1.824±0.019 | 1.940±0.030 | 2.662±0.030 | 0.013±0.002 | 1.402±0.030 | 2.119±0.030 | 2.049±0.030 | 2.453±0.028 | 0.056±0.002 | 1.824±0.024 | 2.743±0.025 | 3.746±0.227 | 3.549±0.023 |
| V2 | *n*-Hexanol | 0.018±0.002 | 0.045±0.002 | 0.018±0.002 | 0.045±0.002 | 0.038±0.002 | 0.017±0.002 | 0.096±0.002 | 0.043±0.002 | 0.101±0.017 | 0.056±0.003 | 0.039±0.001 | 0.298±0.018 | 0.196±0.021 | 0.320±0.024 | 0.221±0.025 |
| V3 | Isobutanol | n.d. | 0.074±0.002 | 0.051±0.003 | 0.060±0.003 | 0.057±0.002 | n.d. | 0.081±0.002 | 0.059±0.002 | 0.101±0.027 | 0.091±0.002 | 0.002±0.000 | 0.138±0.026 | 0.116±0.002 | 0.128±0.026 | 0.119±0.010 |
| V4 | Isoamyl alcohol | 0.015±0.002 | 0.838±0.023 | 0.636±0.003 | 0.982±0.030 | 1.064±0.026 | 0.015±0.002 | 1.253±0.025 | 0.742±0.026 | 1.037±0.026 | 1.301±0.017 | n.d. | 1.934±0.214 | 1.551±0.025 | 1.778±0.229 | 1.995±0.022 |
| V5 | 2-Furanmethanol | 0.026±0.002 | 0.041±0.003 | 0.043±0.002 | 0.046±0.002 | 0.050±0.002 | 0.024±0.002 | 0.046±0.001 | 0.022±0.002 | 0.022±0.001 | 0.025±0.001 | 0.039±0.001 | 0.028±0.002 | 0.024±0.002 | 0.019±0.001 | 0.016±0.001 |
| V6 | 2-phenylethanol | 0.080±0.002 | 1.326±0.234 | 1.146±0.028 | 1.751±0.030 | 1.795±0.030 | 0.077±0.002 | 2.268±0.024 | 1.798±0.166 | 2.689±0.023 | 2.654±0.022 | 0.251±0.002 | 2.760±0.197 | 3.250±0.178 | 2.289±0.025 | 4.017±0.025 |
| **Aldehydes** | | | | | | | | | | | | | | | | |
| V7 | Acetaldehyde | n.d. | 0.031±0.002 | 0.020±0.003 | n.d. | n.d. | 0.042±0.002 | 0.023±0.002 | 0.065±0.002 | n.d. | n.d. | n.d. | 0.018±0.002 | 0.090±0.002 | 0.027±0.003 | n.d. |
| V8 | Benzaldehyde | 0.095±0.002 | 0.033±0.002 | 0.019±0.019 | 0.050±0.002 | 0.055±0.002 | 0.081±0.002 | 0.075±0.001 | 0.026±0.002 | 0.049±0.002 | 0.059±0.002 | 0.273±0.003 | 0.051±0.002 | 0.041±0.002 | 0.027±0.002 | 0.057±0.002 |
| V9 | Prunolide | n.d. | 0.075±0.002 | 0.097±0.003 | 0.144±0.024 | 0.165±0.026 | n.d. | 0.070±0.001 | 0.090±0.002 | 0.132±0.012 | 0.174±0.022 | n.d. | 0.058±0.003 | 0.051±0.002 | 0.096±0.002 | 0.109±0.010 |
| **Esters** | | | | | | | | | | | | | | | | |
| V10 | Ethyl acetate | 0.023±0.002 | 0.766±0.021 | 0.736±0.002 | 0.543±0.023 | 0.779±0.025 | n.d. | 0.385±0.025 | 0.416±0.019 | 0.189±0.022 | 0.360±0.013 | n.d. | 0.587±0.026 | 0.652±0.026 | 0.627±0.025 | 0.669±0.024 |
| V11 | Ethyl butyrate | 0.036±0.002 | 0.439±0.020 | 0.522±0.016 | 0.369±0.021 | 0.813±0.023 | 0.015±0.002 | 0.312±0.022 | 0.604±0.030 | 0.307±0.023 | 0.777±0.030 | n.d. | 0.391±0.023 | 0.369±0.026 | 0.339±0.027 | 0.650±0.025 |
| V12 | Ethyl valerate | n.d. | 0.038±0.002 | 0.028±0.002 | 0.038±0.003 | 0.057±0.002 | n.d. | 0.040±0.002 | 0.029±0.002 | 0.020±0.002 | 0.051±0.002 | n.d. | 0.040±0.003 | 0.023±0.003 | n.d. | 0.041±0.002 |
| V13 | Ethyl caproate | 1.259±0.030 | 8.428±0.207 | 5.102±0.257 | 8.060±0.242 | 10.934±0.030 | 0.828±0.030 | 7.812±0.259 | 4.687±0.234 | 5.661±0.030 | 8.758±0.030 | 1.512±0.022 | 8.019±0.247 | 4.695±0.228 | 5.981±0.026 | 7.651±0.254 |
| V14 | Ethyl lactate | 0.102±0.026 | 0.135±0.003 | 0.131±0.019 | 0.277±0.023 | 0.353±0.025 | 0.090±0.003 | 0.119±0.030 | 0.121±0.029 | 0.125±0.025 | 0.410±0.025 | 0.217±0.002 | 0.087±0.002 | 0.071±0.002 | 0.247±0.025 | 0.266±0.024 |
| V15 | Ethyl heptanoate | 0.016±0.002 | 0.229±0.029 | 0.107±0.015 | 0.167±0.026 | 0.205±0.003 | 0.011±0.002 | 0.206±0.025 | 0.099±0.003 | 0.137±0.031 | 0.165±0.023 | 0.026±0.002 | 0.173±0.028 | 0.053±0.003 | 0.179±0.024 | 0.065±0.005 |
| V16 | Ethyl octanoate | 0.013±0.003 | 0.509±0.031 | 0.276±0.028 | 0.543±0.027 | 0.790±0.051 | 0.013±0.003 | 0.755±0.028 | 0.343±0.038 | 0.525±0.027 | 0.904±0.026 | 0.032±0.003 | 0.646±0.056 | 0.364±0.042 | 0.657±0.015 | 0.807±0.040 |
| V17 | Ethyl Nonanate | 0.008±0.003 | 0.277±0.017 | 0.190±0.029 | 0.362±0.038 | 0.541±0.040 | 0.051±0.001 | 0.489±0.032 | 0.237±0.045 | 0.353±0.012 | 0.533±0.024 | n.d. | 0.446±0.028 | 0.319±0.026 | 0.460±0.039 | 0.507±0.011 |
| V18 | Ethyl caprate | n.d. | 0.104±0.016 | 0.104±0.028 | 0.224±0.013 | 0.323±0.021 | n.d. | 0.215±0.026 | 0.090±0.002 | 0.329±0.003 | 0.498±0.027 | n.d. | 0.222±0.033 | 0.205±0.029 | 0.373±0.042 | 0.438±0.030 |
| V19 | Ethyl dodecanoate | n.d. | 0.671±0.031 | 0.534±0.029 | 0.531±0.019 | 0.602±0.026 | n.d. | n.d | n.d | 0.490±0.0220 | 0.566±0.022 | n.d. | 0.418±0.010 | 0.336±0.026 | 0.379±0.030 | 0.640±0.030 |
| V20 | Ethyl tetradecanoate | 0.032±0.002 | 0.791±0.031 | 0.803±0.031 | 0.924±0.030 | 0.979±0.025 | 0.026±0.002 | 0.780±0.002 | 1.048±0.197 | 1.177±0.026 | 1.163±0.025 | 0.155±0.017 | 0.790±0.036 | 0.970±0.031 | 1.207±0.031 | 1.183±0.026 |
| V21 | Ethyl palmitate | 0.013±0.002 | 9.456±0.273 | 10.775±0.310 | 11.298±0.202 | 12.462±0.285 | 0.426±0.014 | 11.214±0.031 | 13.234±0.239 | 14.874±0.663 | 15.015±0.250 | 6.058±0.030 | 12.519±0.228 | 14.965±0.187 | 16.543±0.092 | 16.486±0.243 |
| V22 | Ethyl linoleate | 0.454±0.024 | 4.841±0.329 | 6.445±0.118 | 6.180±0.249 | 5.982±0.091 | 0.440±0.022 | 4.861±0.290 | 6.959±0.014 | 3.729±0.403 | 2.902±0.326 | 4.360±0.226 | 4.917±0.302 | 7.460±0.300 | 0.205±0.028 | 3.453±0.033 |
| V23 | (E)- Ethyl Oleate | 0.251±0.027 | 3.495±0.213 | 4.944±0.362 | 5.283±0.298 | 5.039±0.312 | 0.240±0.028 | 3.595±0.283 | 5.252±0.221 | 6.029±0.305 | 5.348±0.316 | 4.139±0.060 | 3.769±0.197 | 5.780±0.233 | 4.103±0.131 | 3.200±0.137 |
| V24 | Ethyl benzoate | n.d. | 0.123±0.012 | 0.181±0.030 | 0.325±0.034 | 0.472±0.006 | n.d. | 0.141±0.002 | 0.164±0.033 | 0.316±0.007 | 0.483±0.018 | n.d. | 0.126±0.019 | 0.203±0.018 | 0.366±0.018 | 0.474±0.017 |
| V25 | Phenethyl acetate | 0.009±0.000 | 0.069±0.002 | 0.098±0.003 | 0.171±0.006 | 0.222±0.003 | n.d. | 0.079±0.002 | 0.096±0.027 | 0.106±0.011 | 0.251±0.007 | 0.034±0.003 | 0.083±0.001 | 0.091±0.003 | 0.224±0.014 | 0.270±0.009 |
| V26 | Ethyl 3-phenylpropanoate | n.d. | 0.240±0.036 | 0.256±0.031 | 0.328±0.027 | 0.345±0.025 | n.d. | 0.204±0.027 | 0.087±0.003 | 0.279±0.027 | 0.306±0.028 | n.d. | 0.251±0.010 | 0.229±0.030 | 0.198±0.028 | 0.283±0.010 |
| V27 | Pentanoic acid, 2-hydroxy-4-methyl-,ethyl ester | 0.053±0.004 | 0.079±0.002 | 0.123±0.028 | 0.268±0.006 | 0.282±0.005 | n.d. | 0.080±0.002 | 0.092±0.003 | 0.183±0.022 | 0.157±0.031 | 0.148±0.026 | 0.063±0.003 | 0.189±0.002 | 0.213±0.022 | 0.318±0.017 |
| V28 | Phenethyl acetate | 0.015±0.002 | 0.078±0.003 | 0.061±0.003 | 0.089±0.001 | 0.083±0.003 | n.d. | 0.055±0.002 | 0.041±0.002 | 0.058±0.003 | 0.049±0.002 | n.d. | 0.234±0.001 | 0.062±0.005 | 0.194±0.025 | 0.136±0.003 |
| V29 | Ethyl 9-hexadecenoate | 0.025±0.003 | 0.294±0.029 | 0.376±0.023 | 0.327±0.028 | 6.602±0.327 | 0.022±0.003 | 0.265±0.036 | 0.412±0.001 | 0.506±0.122 | 0.494±0.021 | 0.154±0.016 | 0.297±0.021 | 1.525±0.028 | 0.400±0.027 | 0.404±0.030 |
| V30 | Methyl palmitate | 0.032±0.003 | 0.018±0.001 | 0.028±0.003 | 0.020±0.002 | 0.023±0.002 | 0.017±0.003 | 0.048±0.002 | 0.033±0.002 | 0.033±0.004 | 0.049±0.003 | 0.058±0.002 | 0.054±0.003 | n.d. | 0.023±0.003 | 0.040±0.002 |
| V31 | ç-Methyl linolenate | n.d. | n.d. | n.d. | 0.093±0.002 | 0.098±0.002 | n.d. | n.d. | n.d. | 0.085±0.002 | n.d. | n.d. | n.d. | n.d. | 0.190±0.031 | n.d. |
| V32 | 3-Methylbutyl butyrate | n.d. | 0.028±0.002 | 0.025±0.002 | 0.027±0.001 | 0.036±0.001 | n.d. | n.d. | n.d. | 0.038±0.002 | 0.036±0.002 | n.d. | 0.052±0.003 | 0.023±0.003 | 0.028±0.002 | 0.040±0.003 |
| V33 | Diethyl succinate | 0.019±0.002 | 0.026±0.001 | 0.078±0.002 | 0.249±0.040 | 0.235±0.020 | 0.016±0.002 | 0.048±0.002 | 0.081±0.002 | 0.387±0.019 | 0.430±0.029 | 0.048±0.002 | 0.065±0.003 | 0.056±0.002 | 0.240±0.021 | 0.287±0.006 |
| V34 | Diethyl suberate | n.d. | n.d. | n.d. | 0.045±0.002 | 0.044±0.002 | n.d. | n.d. | n.d. | 0.049±0.002 | 0.049±0.002 | n.d. | n.d. | n.d. | 0.079±0.003 | n.d. |
| V35 | Diethyl azelate | 0.009±0.000 | 0.077±0.001 | 0.100±0.012 | 0.126±0.019 | 0.106±0.015 | n.d. | 0.105±0.003 | 0.067±0.002 | 0.279±0.036 | 0.258±0.028 | 0.051±0.003 | 0.241±0.010 | 1.470±0.036 | 0.380±0.031 | 0.359±0.029 |
| V36 | Isobutyl caproate | n.d. | 0.034±0.002 | n.d. | n.d. | 0.018±0.002 | n.d. | 0.041±0.002 | n.d. | n.d. | 0.016±0.002 | n.d. | 0.054±0.002 | 0.132±0.004 | n.d. | n.d. |
| V37 | Isoamyl acetate | n.d. | 0.073±0.003 | n.d. | 0.046±0.002 | 0.058±0.002 | n.d. | 0.036±0.003 | n.d. | n.d. | n.d. | n.d. | 0.163±0.015 | 0.040±0.004 | 0.044±0.003 | 0.082±0.003 |
| V38 | Isoamyl caproate | n.d. | 0.193±0.030 | 0.115±0.014 | 0.214±0.005 | 0.302±0.029 | n.d. | 0.253±0.025 | 0.127±0.022 | 0.167±0.027 | 0.291±0.013 | n.d. | 0.400±0.032 | 0.218±0.030 | 0.305±0.020 | 0.235±0.029 |
| V39 | ε-Decalactone | 0.007±0.000 | 0.219±0.001 | 0.254±0.001 | 0.292±0.026 | 0.339±0.003 | n.d. | 0.224±0.008 | 0.322±0.027 | 0.325±0.022 | 0.405±0.007 | 0.063±0.001 | 0.517±0.019 | 0.614±0.033 | 0.705±0.032 | 0.199±0.031 |
| **Acids** | | | | | | | | | | | | | | | | |
| V40 | Butanoic acid | 0.080±0.003 | 0.019±0.003 | n.d. | n.d. | n.d. | 0.058±0.003 | 0.031±0.002 | n.d. | n.d. | n.d. | n.d. | n.d. | n.d. | n.d. | n.d. |
| V41 | Hexanoic acid | n.d. | 0.447±0.004 | n.d. | 0.016±0.002 | n.d. | n.d. | n.d. | n.d. | 0.250±0.277 | 0.286±0.046 | n.d. | 0.154±0.029 | 0.091±0.023 | 0.199±0.025 | 0.227±0.021 |
| **Penols** | | | | | | | | | | | | | | | | |
| V42 | Guaiacol | 1.179±0.234 | 0.209±0.025 | 0.184±0.026 | 0.248±0.005 | 0.239±0.028 | 1.167±0.178 | 0.266±0.027 | 0.207±0.027 | 0.275±0.007 | 0.269±0.005 | 3.564±0.234 | 0.250±0.032 | 0.206±0.029 | 0.263±0.013 | 0.266±0.018 |
| V43 | 4-Ethylguaiacol | 0.012±0.003 | 0.036±0.002 | 0.035±0.002 | 0.062±0.002 | 0.044±0.002 | n.d. | 0.071±0.002 | 0.057±0.002 | 0.086±0.026 | 0.073±0.002 | 0.031±0.002 | 1.014±0.044 | 0.681±0.030 | 0.911±0.034 | 0.841±0.032 |
| V44 | 2-Eethoxy-4-vinylphenol ethyl guaiacol | 0.116±0.022 | 6.911±0.370 | 6.675±0.454 | 7.735±0.303 | 7.339±0.258 | 0.104±0.012 | 1.134±0.034 | 2.439±0.019 | 2.710±0.267 | 2.457±0.208 | 0.866±0.022 | 2.112±0.011 | 1.907±0.147 | 2.287±0.203 | 2.325±0.147 |

n.d., not detected.

All the results are the average and error of triplicate experiments.
